# High-Temperature Behavior and Mechanical Performance of Ceramic Brick and Metakaolin Waste Based-Geopolymer Binder

**DOI:** 10.3390/ma19183890

**Published:** 2026-09-12

**Authors:** Martynas Statkauskas, Danutė Vaičiukynienė, Audrius Grinys, Laura Vitola

**Affiliations:** 1 Faculty of Civil Engineering and Architecture, Kaunas University of Technology, Studentu St. 48, LT-51367 Kaunas, Lithuania; danute.vaiciukyniene@ktu.lt (D.V.); audrius.grinys@ktu.lt (A.G.); laura.vitola_1@rtu.lv (L.V.); 2 Institute of Sustainable Building Materials and Engineering Systems, Riga Technical University, Kipsalas 6A, LV-1048 Riga, Latvia

**Keywords:** geopolymer binder, high-temperature behavior, drying shrinkage, thermal shrinkage, residual mechanical properties

## Abstract

The cement industry is a major source of global CO_2_ emissions, driving the development of low-carbon alternatives such as geopolymers. This study examines geopolymer binders produced from ceramic brick waste (CBW) and metakaolin waste (MKW), evaluating their fresh and hardened properties as well as their performance under elevated temperatures. Five binder compositions were formulated by progressively replacing CBW with MKW (25–100 wt.%). The alkaline activator ratio (Na_2_SiO_3_/NaOH = 1.5) and NaOH molality (8 M) were kept constant. Fresh-state behavior was evaluated using Suttard viscometry and Vicat testing, while hardened-state performance was assessed through compressive and flexural strength, softening coefficient, and drying shrinkage. Thermal resistance was examined at 200, 400, 600, and 800 °C, supported by XRD, FT IR, and SEM analyses. The present study investigates how waste-derived aluminosilicate precursors with differing crystallinity and reactivity affect geopolymerization mechanisms and high-temperature phase evolution. MKW-rich binders were found to form highly reactive amorphous gels, resulting in superior early mechanical strength, whereas CBW-rich binders retained thermally stable crystalline phases that enhanced resistance to structural degradation at elevated temperatures. The MKW-rich formulation (F5) demonstrated the highest ambient mechanical performance, reaching 82.8 MPa after curing at 200 °C, due to intensified secondary geopolymerization. In contrast, the CBW-only binder (F1) exhibited superior thermal stability, maintaining a compressive strength of 46.4 MPa even after exposure to 800 °C. These findings establish a clear structure–property relationship between precursor mineralogy, gel chemistry, and high-temperature performance, offering valuable insights for the tailored design of waste-derived geopolymers with optimized thermal and mechanical properties.

## 1. Introduction

The accelerated urban development that has taken place in recent years has resulted in a substantial increase in Portland cement consumption. Portland cement is the primary constituent of concrete, the most widely utilized construction material on a global scale. Recent data indicate that global cement production demonstrated a marked increase from 1.60 billion tonnes in 2000 to 4.20 billion tonnes in 2020 [1]. Nevertheless, the production of cement has been demonstrated to have considerable environmental impacts due to the consumption of natural resources, the utilization of substantial amounts of energy, and the resultant emission of CO_2_ [2]. The emission of approximately 0.95 tonnes of CO_2_ for every tonne of cement produced is well documented, with most of these emissions occurring during the clinker production process [3]. It is estimated that cement production contributes approximately 5–8% of global anthropogenic CO_2_ emissions [4]. This has led to increased demand for more sustainable alternatives. One approach to reducing the environmental impact of cement production is to reduce the clinker content of cement or to replace Portland cement with alternative binders produced from waste and industrial by-products. In this context, significant attention has been given to alkali-activated materials (AAMs) as potential alternatives to Portland cement [5].

Alkali activation is defined as the reaction of an aluminosilicate precursor with an alkaline activator, forming a hardened binder [6]. AAMs are typically categorized as either high-calcium systems, such as those based on blast furnace slag, iron ore tailing and Class C fly ash, or low-calcium systems, such as those based on metakaolin and Class F fly ash. Low-calcium systems are commonly referred to as geopolymers. Geopolymers were first developed by Joseph Davidovits [7] and are regarded as a promising alternative to conventional cementitious materials. These materials are characterized as inorganic polysilicate-aluminate compounds, formed from aluminum- and silicon-rich sources [8]. The production process typically involves the utilization of alkaline activators, such as KOH or NaOH, followed by the imposition of suitable curing conditions. The geopolymerization process comprises precursor dissolution, reorganization, condensation, and polymerization [5,6,7,8,9]. It is hypothesized that the optimal composition of alkali-activated materials has the potential to engender a decline in greenhouse gas emissions, reaching a maximum of 80% when contrasted with the emissions generated by conventional concrete.

The rheological properties of fresh geopolymer mixtures have been demonstrated to have a significant impact on their workability and hardened properties. Conventional methodologies, encompassing mini slump, viscometer, and penetration tests, are frequently employed for assessment. The Bingham and Herschel–Bulkley models [10] are utilized to ascertain yield stress and viscosity. In comparison with cement paste, geopolymer paste typically exhibits a lower yield stress but higher plastic viscosity [11]. The workability of the material is contingent upon the type of precursor, the composition of the activator, and the water-to-binder ratio [12]. A number of studies have examined these relationships [13,14,15,16]. Kashani et al. [13] established a correlation between the SiO_2_/Al_2_O_3_ ratio and the initial setting time, while Provis et al. [14] and Rovnaník et al. [16] reported the presence of strong thixotropy and high yield stress in metakaolin-based systems. Favier et al. [10] partly attributed the high viscosity to the viscous Na_2_SiO_3_ solution. Consequently, the composition of geopolymer must be taken into consideration when determining workability.

The ability of construction materials to withstand elevated temperatures is a critical consideration. Cementitious materials have been observed to undergo substantial degradation at elevated temperatures [17]. Furthermore, the decomposition of Ca(OH)_2_ has been observed to occur at approximately 400 °C [18], while thermal expansion differences have been shown to induce cracking and result in strength losses of up to 95% at 1000 °C [19]. Concrete may also undergo a process of fire-induced spalling [20]. Geopolymers have been shown to exhibit superior thermal stability, with the capacity to endure temperatures ranging from 1000 to 1200 °C [21]. This enhanced resistance can be attributed to their dense inorganic composition and chemical stability [22], resulting in reduced spalling tendencies [23]. The performance of these materials is contingent upon a number of factors, including the type of precursor, the calcium content, the composition of the activator, the curing regime, the heating and cooling cycles, and the presence of modifiers such as fibers [24].

A substantial body of research [25,26,27,28,29,30] has been conducted on the performance of geopolymers when subjected to high-temperature conditions. Consistent findings were reported by Jinag et al. [25]. Celikten et al. [26] discovered enhanced flexural and compressive strengths at 400 °C in fly ash–sanitaryware waste geopolymer mortars. Albidah et al. [27] reported residual compressive strengths of 56–63%, 38–51%, and 28–34% after exposure to 200, 400, and 600 °C, respectively. Li et al. [28] reported a 25% increase in residual strength after incorporating graphene oxide. Duxson et al. [29] demonstrated that the processes of shrinkage and densification are contingent on the Si/Al ratio and the gel structure. Kong et al. [30] further reported that higher Si/Al ratios have been shown to improve temperature resistance, with only 5% strength loss recorded for ratios above 1.5.

Despite their promising mechanical performance, alkali-activated materials face challenges related to volumetric stability, particularly shrinkage [31]. Shrinkage leads to deformation and cracking, which reduce durability and compromise reinforcement performance [32]. Alkali-activated material shrinkage includes autogenous shrinkage—caused by internal water consumption during reaction—and drying shrinkage, which results from moisture loss to the environment [33]. High-calcium alkali-activated materials typically show greater shrinkage than geopolymers due to rapid reaction kinetics and differences in reaction product volume [34]. Other influencing factors include activator type and dosage, particle size distribution, and curing regime, including curing temperature, relative humidity, and duration [35]. Pore structure also plays a critical role [36]. Heat-curing generally reduces shrinkage by accelerating gel formation and lowering porosity [37,38]. Experimental results demonstrate that shrinkage can be significantly reduced through precursor blending, addition of various aggregates, or partial replacement with fillers such as glass powder [39,40].

Despite the extensive research conducted on ceramic brick waste (CBW)- and metakaolin waste (MKW)-based precursors in alkali-activated systems, the existing studies have predominantly focused on the performance of these materials individually. The high-temperature stability of their combined CBW–MKW composite system remains an area that has received insufficient attention. In particular, the synergistic effects governing phase evolution, microstructural transformation, and residual mechanical performance under elevated temperatures have not yet been systematically clarified. The present study thus seeks to address the research gap identified by comprehensively evaluating the thermo-mechanical behavior and microstructural development of a CBW–MKW geopolymer binder subjected to high-temperature exposure. The objective is to provide new insights into its structural stability and performance enhancement mechanisms.

The aim of this study is to investigate a geopolymer binder produced from recycled ceramic brick waste and metakaolin waste, focusing on its fresh properties (workability, setting time, early reaction kinetics) and hardened characteristics (strength, shrinkage, microstructure) before and after high-temperature exposure. The novelty of this work lies in developing a sustainable, thermally resistant binder created entirely from waste-derived precursors, while examining the relationship between high-temperature performance and mechanical behavior.

## 2. Experimental Section

### 2.1. Experimental Methods

#### 2.1.1. Chemical Composition and Microstructural Parameters

The determination of the precursor elemental composition was accomplished using X-ray fluorescence (XRF). Device: Bruker X-ray S8 Tiger WD spectrometer (Bruker AXS, Karlsruhe, Germany).

The determination of precursor fineness, particle size distribution, and density was conducted using laser diffraction. Device: The CILAS 1090 LD laser scattering particle size analyzer (Cilas, Orléans, France).

The mineralogy of precursor and geopolymer binder hydration products was determined using X-ray diffraction (XRD). Device: The D8 Advance diffractometer, manufactured by Bruker AXS (Bruker AXS, Karlsruhe, Germany), utilizes the Bragg–Brentano geometry. Database (PDF-2) for peak identification. The materials were analyzed quantitatively using the Rietveld method (TOPAS 4.1). The mineralogical validity of geopolymer products of synthesis was verified by employing Fourier transform infrared spectroscopy (FT-IR). Device: Perkin “Elmer FT-IR System” spectrometer (PerkinElmer, Waltham, MA, USA).

The microstructures of precursors and geopolymerization reaction products were examined through the application of scanning electron microscopy (SEM). Device: high-tenacity SEM “FEI Quanta 200 FEG” (FEI, Hillsboro, OR, USA) with a Schottky field emission gun (FEG) (Hitachi, Tokyo, Japan). Chemical composition was investigated with an energy-dispersive X-ray spectroscopy (EDS) detector (Bruker AXS, Karlsruhe, Germany).

#### 2.1.2. Fresh Properties and Heat Evolution

Workability. The rheology of geopolymer binder was determined through a consistency test of fresh geopolymer paste (flow) with a Suttard’s viscometer, in accordance with the method described in the study by Stel’makh et al. [41].

Setting behavior. The alkaline solution to precursor (AS/P) ratio for normal-consistency paste was determined in accordance with the standard EN 196-3 [42]. Furthermore, the initial and final setting time was determined using Vicat apparatus.

Hydration temperature. The evaluation of geopolymer binder hydration temperatures was conducted using the semi-adiabatic calorimetry test procedure, as outlined in EN 196-9 [43]. The laboratory equipment comprises two items: an eight-channel USB TC-08 Data Logger (Bruker AXS, Karlsruhe, Germany) and a K-type thermocouple, which facilitates temperature measurement within a range of −270 °C to +1300 °C.

#### 2.1.3. Mechanical Performance

Strength behavior. The evaluation of the mechanical properties of hardened geopolymer binders was conducted through the implementation of compressive and flexural strength performance tests, in accordance with the established standards EN 12390-3 [44] and EN 12390-5 [45]. The testing of specimens was conducted after 7, 28, and 90 days of curing. Device: computerized hydraulic press (load rate 0.6 ± 0.2 MPa/s). Residual strengths were subsequently evaluated following exposure to elevated temperatures for a designated period. For each geopolymer composition and curative condition, the experimental design comprised five replicate specimens, ensuring statistical reliability. The compressive and flexural strength values that have been reported are shown to be in accordance with the arithmetic mean of the relevant measurements. The dispersion of the results was quantified by calculating the standard deviation. The resultant values indicated that the coefficient of variation remained within acceptable limits (<10%), thereby confirming the consistency and reproducibility of the experimental data.

Water resistance. The durability of geopolymer binder when exposed to water was examined using water absorption and softening coefficient indexes. The softening coefficient is defined as the ratio between the compressive strength (CS) of a saturated specimen and that of a dry specimen. The following Equation (1) is used to express this concept:

(1) $$K=\frac{C_{w}}{C_{d}};$$

where $C_{w}$—CS value after soaking in water for 24 h; $C_{d}$—CS value after 24 h drying.

The water absorption is the ratio of the water absorbed to the dry specimen, established by the following Equation (2):

(2) $$W_{a}=\frac{M_{i}-M}{M}\times 100\%;$$

where $M_{i}$—weight of specimen after 24 h of saturating in water; M—weight of specimen after drying to constant mass.

Shrinkage behavior. The volumetric stability of the geopolymer binders was assessed by measuring drying shrinkage in accordance with standard EN 12390-16 [46]. The length of the geopolymer binder prisms was measured after 3, 7, 14, 28, 56, and 90 days of curing. Drying shrinkage strain was calculated according to the following Equation (3):

(3) $$\varepsilon_{cs}(t,t_{0})=\frac{l{(t}_{0})-l_{cs}(t)}{L_{0}};$$

where $\varepsilon_{cs}(t,t_{0})$—total shrinkage strain of the specimen at the time t; $L_{0}$—gauge length; $l{(t}_{0})$—initial length at the time $t_{0}$; $l_{cs}(t)$—length at time t.

Also, geopolymer prism weights were evaluated after 3, 7, 14, 28, 56 and 90 days of curing, and the change in mass of geopolymer binder was calculated according to the following Equation (4):

(4) $$X_{cs}=\frac{W_{cs}(t)-W(t_{0})}{W(t_{0})};$$

where $X_{cs}$—total change in mass of specimen at the time t; $W_{cs}\left(t\right)$—initial weight at the time t; $W(t_{0})$—weight at time $t_{0}$.

### 2.2. Precursors and Alkaline Activators

**Figure 1:**
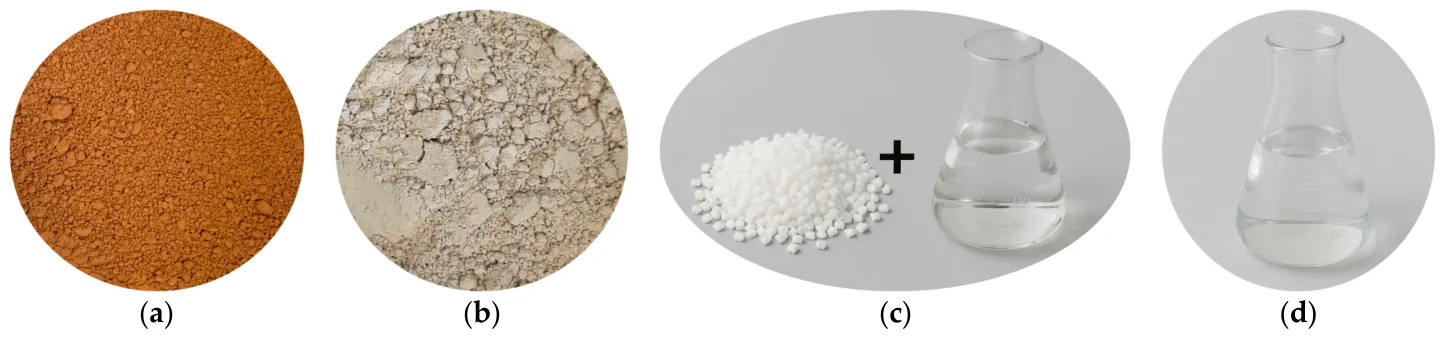
Macroscopic view of the precursors and alkaline activators: (**a**)—ceramic brick waste, (**b**)—metakaolin waste; (**c**)—NaOH solution; (**d**)—Na_2_SiO_3_ solution.

The development of the geopolymer binder system entailed the integration of two precursors: ceramic brick waste (CBW) and metakaolin waste (MKW). CBW (Figure 1a) represents a particularly widespread form of construction and demolition waste. In this experiment, varying sizes of ceramic bricks (up to 30 cm) were crushed into smaller particles measuring between 1.0 and 3.0 cm. Subsequently, the fractured fragments of the bricks were subjected to a milling process that lasted for a period of 2 h. This procedure was undertaken to yield a powder that possessed an optimal specific surface area. Metakaolin, an industrial by-product, is a different phase calcinated form of kaolinite clay, functioning as an anti-agglomeration agent during the manufacturing process of expanded glass granules. MKW (Figure 1b) has a distinct oxide composition (an elevated content of Na_2_O) due to contamination with expanded glass particles when compared with standard metakaolin.

The geopolymer binder was composed of two alkaline activators: sodium hydroxide (NaOH) solution and sodium silicate (Na_2_SiO_3_) solution. The NaOH solution (Figure 1c) was prepared by combining sodium hydroxide pellets (97% purity) with a specified volume of distilled water, yielding a solution concentration of 8 M. The Na_2_SiO_3_ solution (Figure 1d) was procured from a local chemical compounds supplier with a silica modulus (M_s_) of 3.0 (Na_2_O: 9.3%, SiO_2_: 28.0%,) and a specific gravity of 1.38 g/cm^3^.

The elemental composition of the precursor material was evaluated through the implementation of an XRF experimental method. The results of this evaluation have been previously published in our previous study [47] and are outlined in Table 1. XRF results have indicated that the oxide composition of ceramic brick waste is predominantly SiO_2_ (70.49%) and Al_2_O_3_ (13.24%), while metakaolin waste is chiefly SiO_2_ (55.28%), Al_2_O_3_ (30.90%) and Na_2_O (7.46%). These precursor materials are characterized by a substantial presence of amorphous SiO_2_ and Al_2_O_3_, a prerequisite for effective geopolymerization reactions.

Furthermore, the precursor materials were examined through the XRD experimental method to evaluate their mineralogy. These results have been previously published in our study [47] and are summarized in Table 2. A comparison of the precursor materials reveals that both exhibit high concentrations of calcinated SiO_2_ and Al_2_O_3_; however, metakaolin waste is more reactive due to its significantly higher amorphous phase content, which reaches 73.07%.

**Figure 2:**
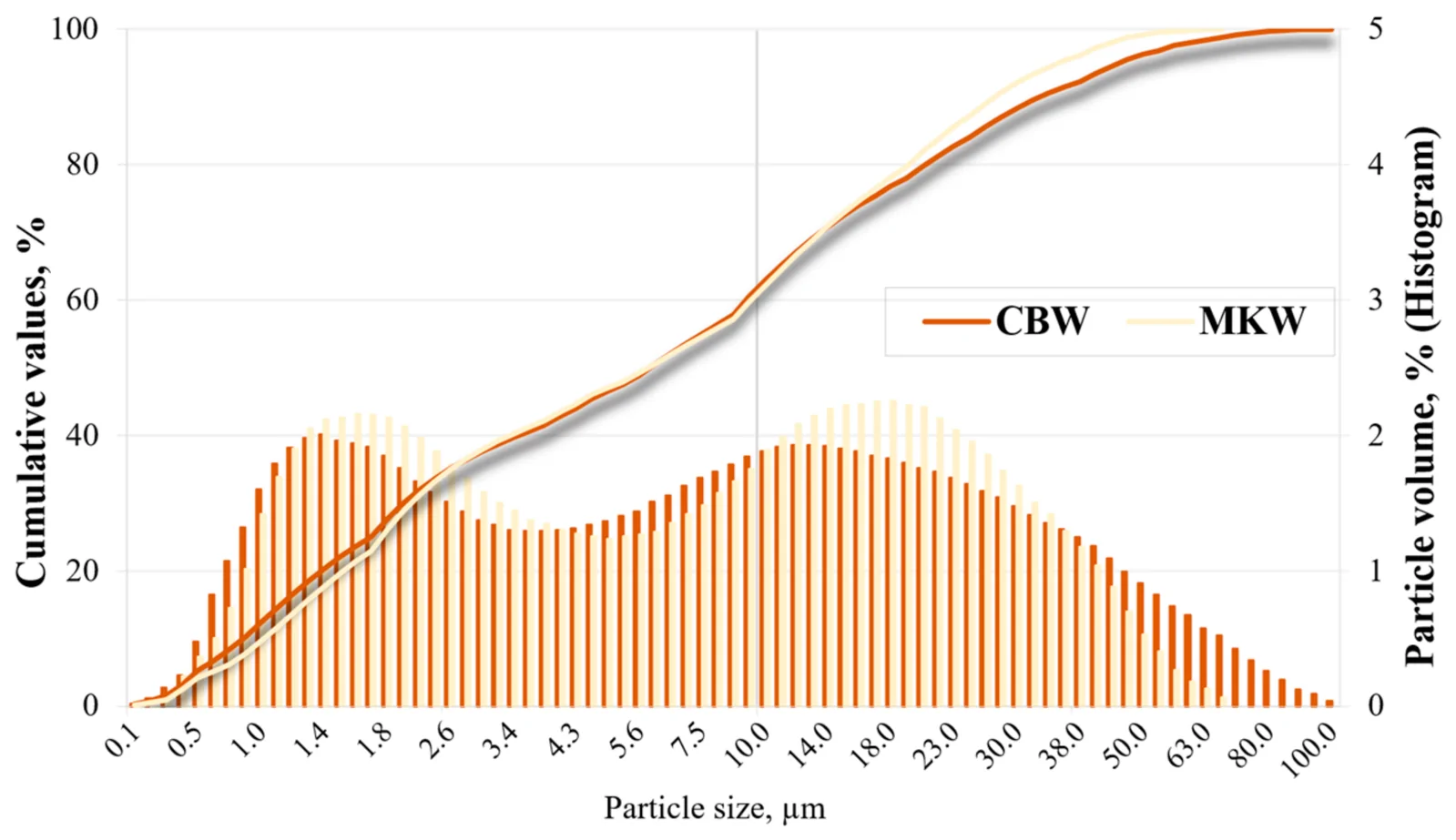
Precursor materials particle size distribution.

The characterization of precursor material particles was performed by means of the laser diffraction method (see Figure 2). Ceramic brick waste (CBW) has a mean particle diameter of 12.89 µm, the highest number of uniform particles (2.01%) at 1.4 µm, particle size distribution in the range of 0.1–100 µm, a density of 2.77 g/cm^3^, and a specific surface area of 497.96 m^2^/kg. Metakaolin waste (MKW) has a mean particle diameter of 11.31 µm, the highest number of uniform particles (2.26%) at 18.0 µm, particle size distribution in the range of 0.1–66 µm, a density of 2.59 g/cm^3^, and a specific surface area of 472.75 m^2^/kg.

**Figure 3:**
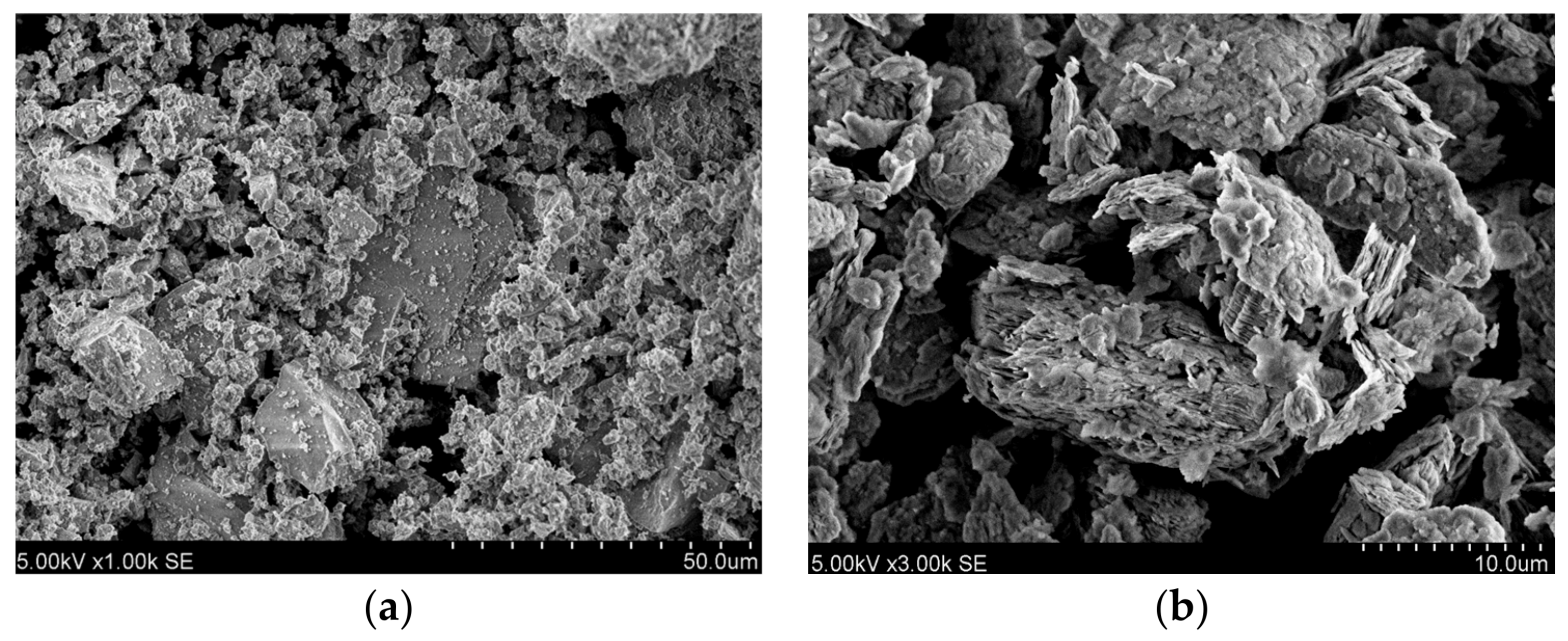
The microstructure of precursor materials: (**a**)—CBW, (**b**)—MKW.

The particle microscopic analysis was conducted using the scanning electron microscopy experimental method (see Figure 3). The ceramic brick waste (CBW) exhibited a sharp-edged particle shape, while metakaolin waste demonstrated a plate-like stratified particle shape.

### 2.3. Mix Design and Specimen Preparation

#### 2.3.1. Geopolymer Compositions

The composition of geopolymer binder mixtures was meticulously devised, with consideration given to the type and fineness of precursor materials, the type and content of the alkaline activator solution, and the SiO_2_/Na_2_O/Al_2_O_3_ ratios. The design of the mixture was based on varying proportions and combinations of ceramic brick (CBW) and metakaolin waste (MKW) in the geopolymer binder. Consequently, five geopolymer binder mixture compositions (F1–F5) were formulated, comprising varying proportions of the aforementioned precursor materials and alkaline activator solutions, as delineated in Table 3. The initial precursor, CBW, was gradually replaced (25, 50, 75 and 100 wt.%) by MKW. The alkaline activator solutions employed in this study were sodium silicate and sodium hydroxide (Na_2_SiO_3_/NaOH ratio—1.5). The sodium hydroxide solution was prepared immediately prior to the geopolymer mixing procedure, wherein NaOH pellets were dissolved in a predetermined quantity of distilled water to achieve a solution molality of 8 M. Subsequent to this, as sodium hydroxide solutions are known to have an exothermic reaction, the solution was permitted to attain room temperature before its utilization in the geopolymer mixing procedure. The findings of the present study [48] demonstrate that an increase in sodium hydroxide dosage is a crucial factor in achieving enhanced geopolymerization reaction efficiency, particularly when elevated levels of metakaolin residue are present within the geopolymer binder composition. The accelerated reaction rate of metakaolin waste compared to ceramic brick waste can be attributed to its higher concentration of Al_2_O_3_ and its greater content of amorphous phase. These characteristics enable a greater number of components to react more rapidly in the initial stage of geopolymer curing. Therefore, the alkaline solution content exhibited variation across all mixture compositions until the geopolymerization reaction reached a state of optimal efficiency. Concurrently, the fresh mixture’s consistency was designed to remain uniform across all compositions. It is noteworthy that the fresh geopolymer binder mixture was not subjected to the addition of supplementary water for consistency adjustment.

#### 2.3.2. Mixing, Casting and Curing Procedures

The geopolymer binder mixing procedure was conducted with an electric mixer (Philips HR3740/00, Philips, Kaunas, Lithuania), with a power output of 450 watts. The initial phase of the mixing procedure entailed the synthesis of two alkaline activators, which were amalgamated for a duration of 1 min. Concurrently, a process known as dry mixing was executed, wherein two precursor materials were mixed. The next step was a three-minute wet mixing process during which a liquid alkaline solution was added to the dry precursors. The total mixing time was recorded as an exact 4 min. The homogeneously mixed geopolymer paste was poured into two types of molds: cubic silicone (40 mm × 40 mm × 40 mm) for compressive strength and softening coefficient tests, and prismatic metal molds (160 mm × 40 mm × 40 mm) for the drying shrinkage and flexural strength tests. Geopolymer paste mixtures were meticulously poured into the molds and subsequently compacted on a vibrating table in two layers to avoid the formation of air bubbles. To mitigate moisture loss during the early stages of the curing process, the molds were coated with polypropylene film. Subsequent to specimen formation, the specimens of geopolymer binder were placed in an environmental setting at ambient temperature (20 ± 2 °C, 45% relative humidity) for a period of 24 h, allowing them to rest. After a rest period of 24 h, a furnace-curing regime was employed (60 °C for 24 h). The aim of this process was to enhance the early strength of the geopolymer binder. The later-curing period continued under the aforementioned ambient temperature conditions until 7, 28, and 90 days had elapsed, at which time mechanical tests were conducted to assess specimen mechanical properties. All mechanical tests were conducted under laboratory ambient conditions of 20 ± 2 °C and around 45% relative humidity.

#### 2.3.3. Heating and Cooling Regime

**Figure 4:**
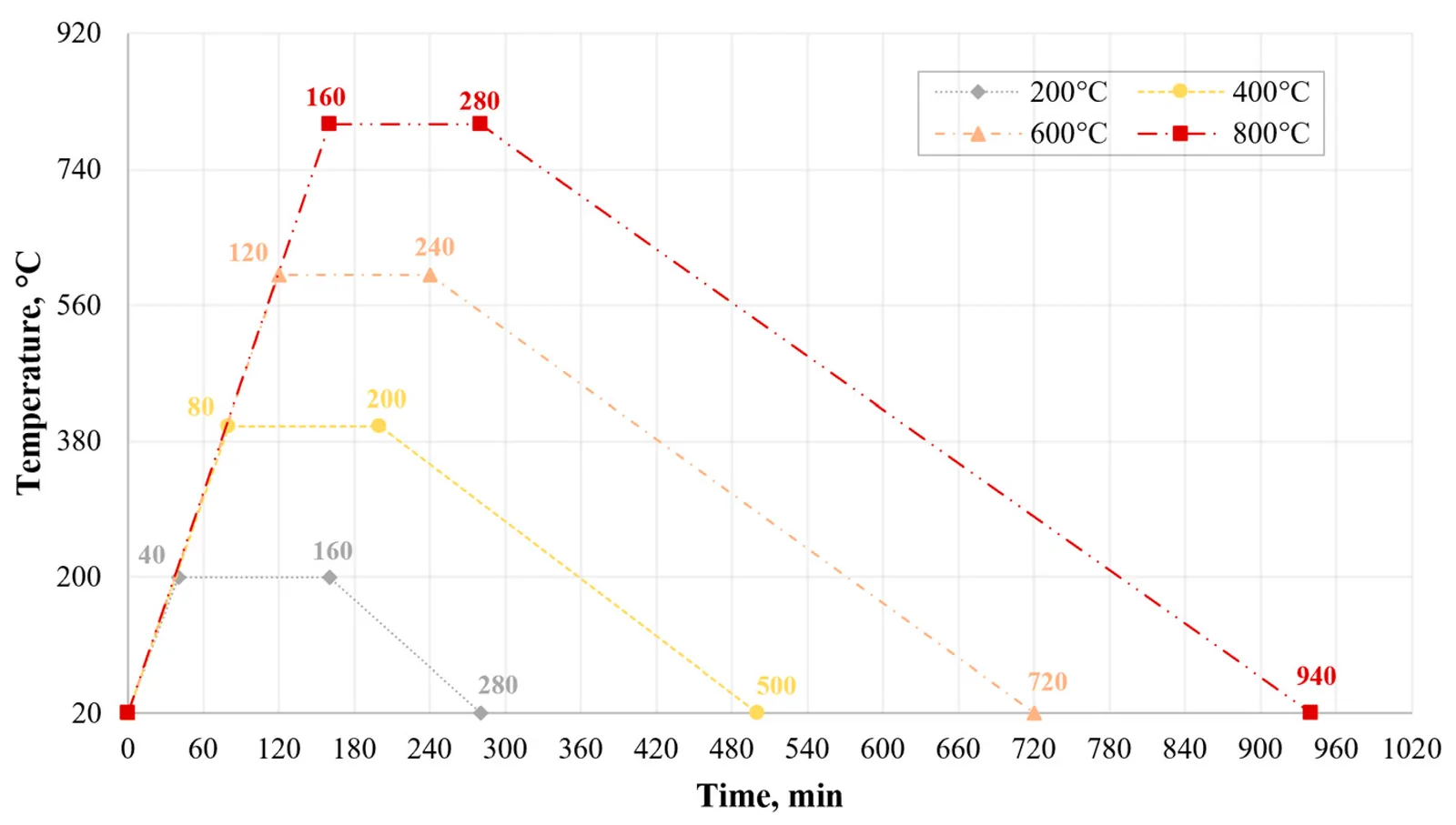
Applied temperature schedules for binder testing at target exposures of 200, 400, 600 and 800 °C.

The elevated temperature regime was modeled using the constant heating rate method. The selection of this method was motivated by the objective of rapidly attaining the targeted temperature. The temperature of the system was increased from the ambient level to the desired temperatures of 200, 400, 600, and 800 °C at a rate of 5 °C/min using an electric furnace (Figure 4). To ensure the uniform distribution of heat across the specimens, the targeted temperature was sustained for a duration of 2 h [49]. The cooling method employed in this study was a natural (air) cooling process within the furnace. The selection of this method was predicated on considerations of safety. The geopolymer specimens were subjected to gradual cooling in a furnace until they attained ambient room temperature. Later, a 24 h repose period was initiated prior to conducting the subsequent evaluation, focusing on the post-heating characteristics (residual strength and thermal shrinkage) of the geopolymer specimens.

## 3. Results and Discussion

### 3.1. Fresh-State Properties

#### 3.1.1. Consistency and Setting Time

**Figure 5:**
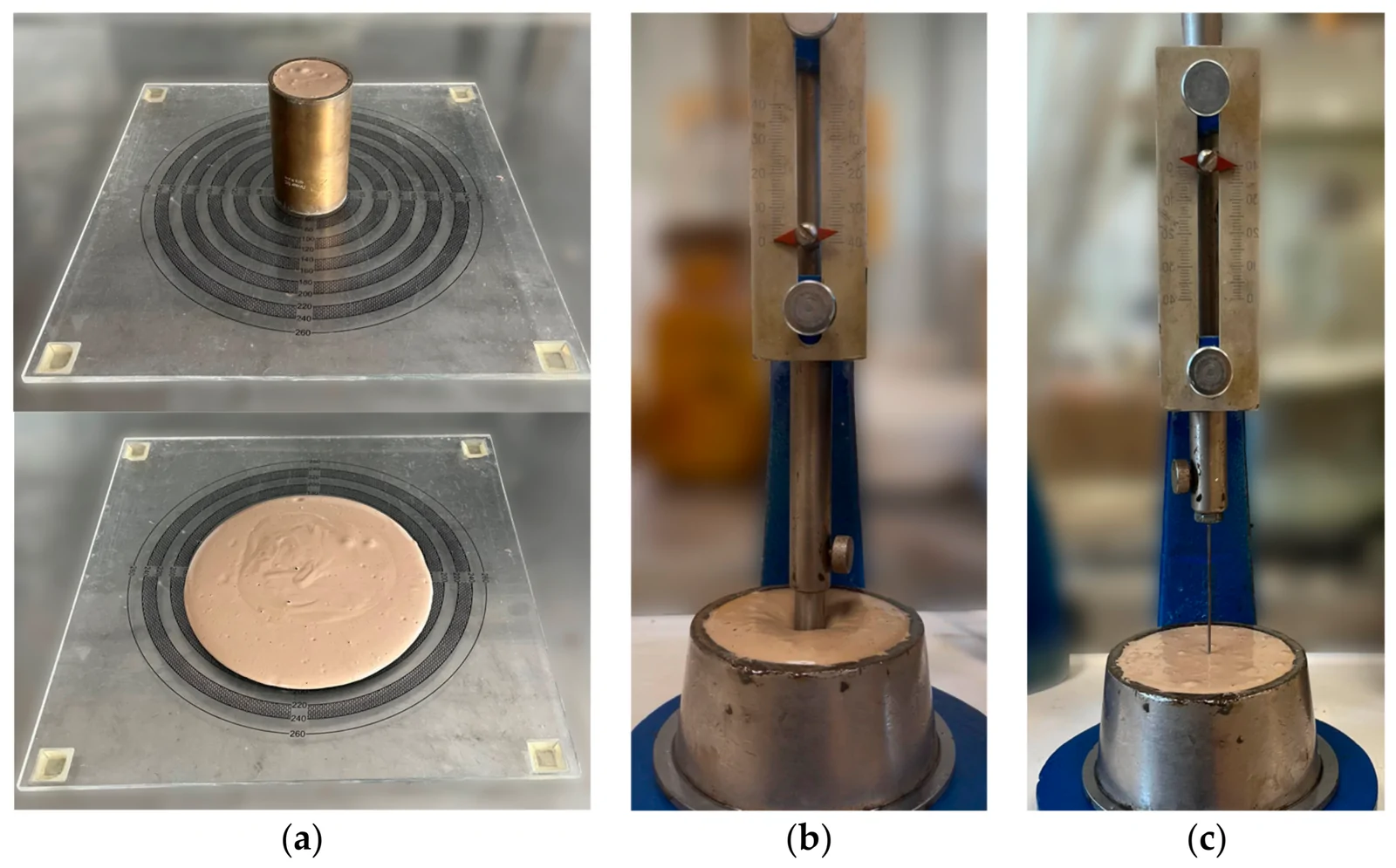
Fresh-state geopolymer properties: (**a**)—flowability (Suttard viscometer); (**b**)—consistency (Vicat plunger); (**c**)—setting time (Vicat needle).

The workability of fresh geopolymer paste was evaluated through the implementation of Suttard’s viscometer test. In this experiment, the flowability of geopolymer paste is evaluated immediately post-mixing. The freshly prepared paste was poured into a cylindrical mold and positioned centrally on a graded glass plate, being filled to the top and then gently lifted vertically to evaluate the spread of the mixture (see Figure 5a). The consistency (flowability) of the ceramic brick and metakaolin waste-based geopolymer paste was planned to be in the range of 180 ± 10 mm for every designed geopolymer composition, in similarity to the study by Krivenko et al. [50]. This range was selected to ensure the consistency of the geopolymer mixture in each of the designed compositions, despite the varying quantities of precursor materials and activators, which consequently resulted in different AS/P ratios. The compositions were designed correctly, as it was found that the test results were within the required range (180 ± 10 mm). Furthermore, it was determined that the F4 and F5 compositions exhibited a slightly higher flow spread in comparison to the F1, F2, and F3 compositions (see Table 4).

In addition, an alternative non-standard test method was employed to evaluate the consistency of the mixture across every composition. This method involved the use of the Vicat apparatus with a plunger, which was used to measure the time (seconds) taken for the plunger to reach the bottom of the mold filled with fresh geopolymer mixture (see Figure 5b). The findings of the study have demonstrated that, despite the Vicat plunger times to bottom being similar in the F1–F5 compositions (0.63–0.41 s), the mixtures in which the Vicat plunger reached the bottom of the mold most rapidly (0.41–0.43 s) exhibited slightly higher flowability (190 mm).

The highest fresh-paste density was achieved in the composition with the highest CBW precursor content (F1)—2031 kg/m^3^, while the lowest density was achieved with the highest content of MKW precursor (F5)—1694 kg/m^3^. The geopolymer fresh-paste density demonstrated an upward trend in conjunction with an increase in the quantity of ceramic brick waste. This phenomenon is attributed to the superior particle density, lower porosity and better packing efficiency of ceramic brick waste precursor particles in comparison to metakaolin waste precursor particles.

The initial and final setting times of geopolymer paste were evaluated through the penetration test with the Vicat apparatus and needle (see Figure 5c). The setting behavior was assessed in accordance with the European standard EN 196-3 [42], which stipulates that the initial setting time is defined as the point at which the needle penetrates 6 ± 3 mm from the bottom of the mold, and that the final setting time is reached when it penetrates no more than 0.5 mm. The initial and final setting times of the geopolymer paste are presented in Table 4. The fastest setting behavior was observed in the composition (F1) containing 100% ceramic brick waste precursor and the lowest AS/P ratio, where the initial setting time was 150 min and the final setting time was 205 min. Similar setting times were obtained in the study by Pasupathy et al. [51], where brick waste geopolymer initial setting time varied from 140 to 315 min, and final setting times ranged from 170 to 355 min with varying Na_2_O concentrations. The slowest setting behavior was found in the composition (F5) with 100% metakaolin waste precursor and the highest AS/P ratio, where the initial setting time was 540 min and the final setting time was 600 min. For comparison, the initial setting time of the metakaolin-based geopolymer in the study by Mo et al. [52] was 953 min, and its final setting time was 1336 min. This finding is corroborated by Chen et al. [53,54], who state that it takes 900–1200 min to reach metakaolin-based geopolymer final setting time. Consequently, a discernible tendency has been identified: the incorporation of metakaolin waste has been observed to result in prolonged geopolymer binder setting times. One of the factors that may contribute to the slower setting behavior observed in compositions containing metakaolin waste is the comparatively lower calcium content of the MKW precursor than that of the CBW precursor. As reported by Kim et al. [55], increased calcium content in geopolymers can contribute to accelerated setting. However, the observed setting behavior cannot be attributed to calcium content alone, as other precursor characteristics, including amorphous content, particle morphology, and fineness, may also influence the reaction kinetics and setting time. Another factor affecting the setting time is the alkaline solution-to-precursor (AS/P) ratio. Also, a higher AS/P ratio increases the water content associated with the alkaline solution, which can contribute to a longer setting time. Therefore, the faster setting observed for the CBW-rich composition is likely related to the combined effects of calcium content, precursor characteristics, and the lower AS/P ratio.

Thus, the setting time constitutes a pivotal geopolymer mixture parameter, regulating the duration over which the mixture can be placed and compacted. It is recommended that a meticulous approach be taken to the selection of precursor materials, alkaline activators, their concentration, the Na_2_SiO_3_/NaOH ratio, and the AS/P ratio. These factors have the capacity to exert a positive or negative influence on the setting times of geopolymers [56]. Additionally, Mo et al. [52] have proved that increasing curing temperature (40–100 °C) is a solution to slower metakaolin-based geopolymer setting, as it radically hastens both the initial and final setting times. This phenomenon is attributed to the accelerated polymerization and solidification of the geopolymer pastes within a sealed environment at higher temperatures.

#### 3.1.2. Heat Evolution

**Figure 6:**
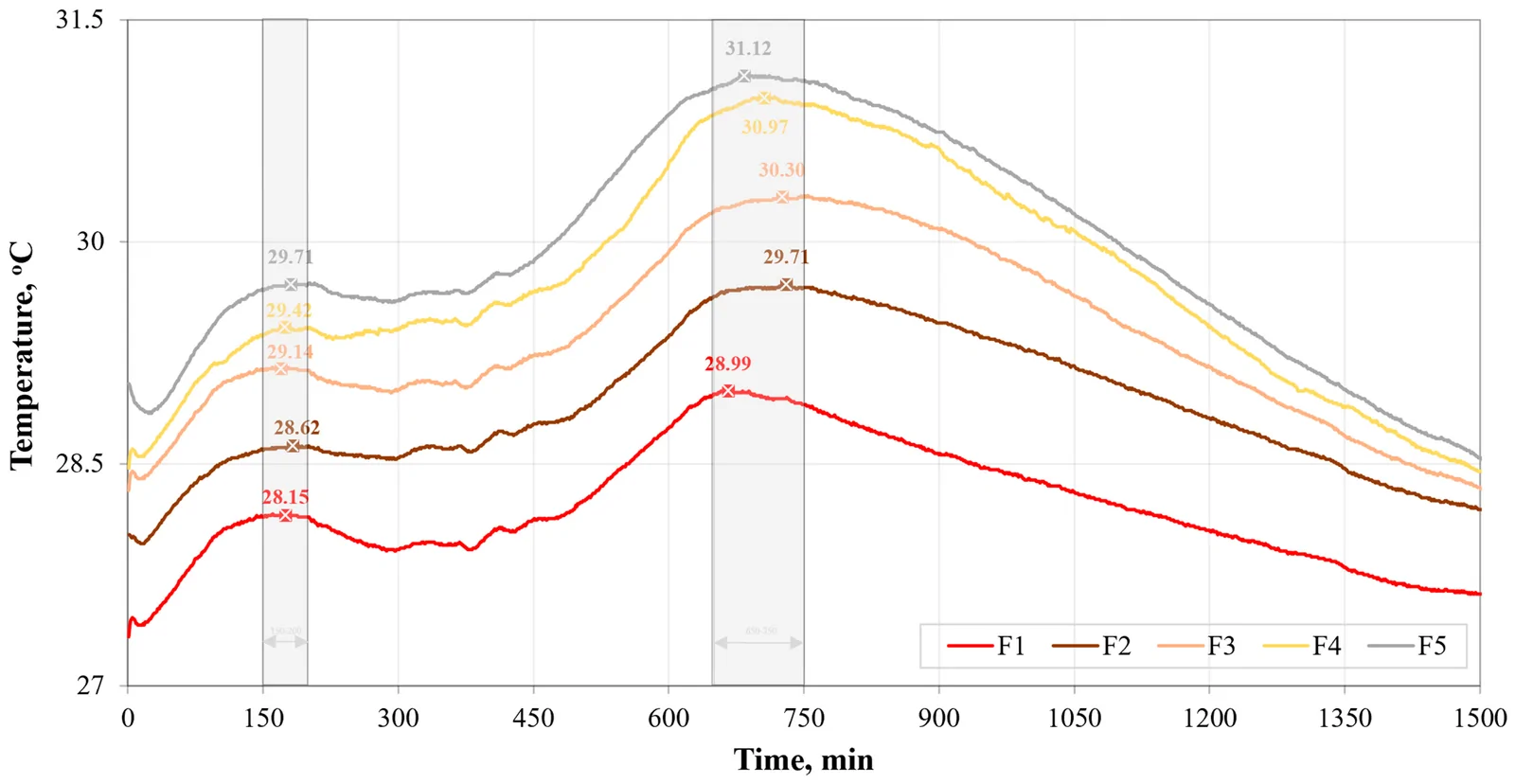
Heat evolution behavior of geopolymer pastes during hydration, as implied by temperature alterations over time.

Geopolymerization is defined as an exothermic reaction that exhibits varying levels of development, contingent on the properties of the precursor and activator materials utilized. It is therefore vital to understand the temporal and quantitative aspects of the heat produced during the geopolymerization reaction. Thus, the heat evolution within the first 24 h of geopolymer paste formation was determined through the implementation of a semi-adiabatic calorimetry test. The heat evolution curves (semi-adiabatic calorimetry) of every geopolymer binder composition (F1–F5) that was designed are presented in Figure 6.

The heat evolution curves demonstrated two peaks, which show a comparable three-stage exothermic process, as previously reported in other studies [57,58,59]. The first peak occurs at approximately 150–200 min, and is responsible for stage I, which is colloquially referred to as ‘destruction’, at which point precursors encountered alkaline activators. This process is responsible for the dissolution of CBW and MKW into silicate and aluminate monomers. The second peak emerges in the range of 650–750 min, which is designated as stage II and is known as ‘polymerization’. This stage is responsible for the geopolymerization process. At this stage, the aluminate and silicate monomers undergo polymerization to form alumino-silicate oligomers and geopolymer fragments, resulting in the formation of a three-dimensional alumino-silicate system (Si-O-Al). This peak is responsible for the early-age strength and mechanical integrity of the geopolymer binder. The III stage is referred to as ‘stabilization’ and is characterized by a decline in the calorimetry curve, which occurs after the second peak (after 750 min). This stage is responsible for the reorganization and stabilization of newly formed gel, which results in long-term strength gain.

The calorimetry curves demonstrated that geopolymer compositions with higher quantities of metakaolin waste exhibited elevated levels of heat, as the maximum heat (31.12 °C) was achieved in composition F5, which contradicts the findings of our previous study, where ceramic brick-based geopolymer had the highest level of heat [48]. In this instance, compositions involving MKW were activated with elevated concentrations of alkaline activators. This approach was adopted due to the high content of soluble SiO_2_ in MKW precursor, a factor that was not fully exploited in our previous study. The concentration of alkaline solutions has been demonstrated to exert an influence on the geopolymerization process, with the reaction heat rate increasing in proportion to the concentration of NaOH or Na_2_SiO_3_ [57]. As in this study, the geopolymer binder compositions were designed to ensure the NaOH concentration of 8 M and the Na_2_SiO_3_/NaOH ratio of 1.5 were constant. The sole ratio that demonstrated variability was that of the alkaline solution to precursor (AS/P), which exhibited an upward trend concomitant with the gradual increase in the quantity of metakaolin waste across the F1–F5 compositions. The increasing quantity of alkaline activators led to more favorable SiO_2_/Al_2_O_3_ and H_2_O/Na_2_O ratios, which are beneficial for the intensified geopolymerization reaction and are responsible for higher heat evolution with ultimately better hardened geopolymer mechanical properties [57,58].

### 3.2. Mechanical Properties

#### 3.2.1. Compressive and Flexural Strength

**Figure 7:**
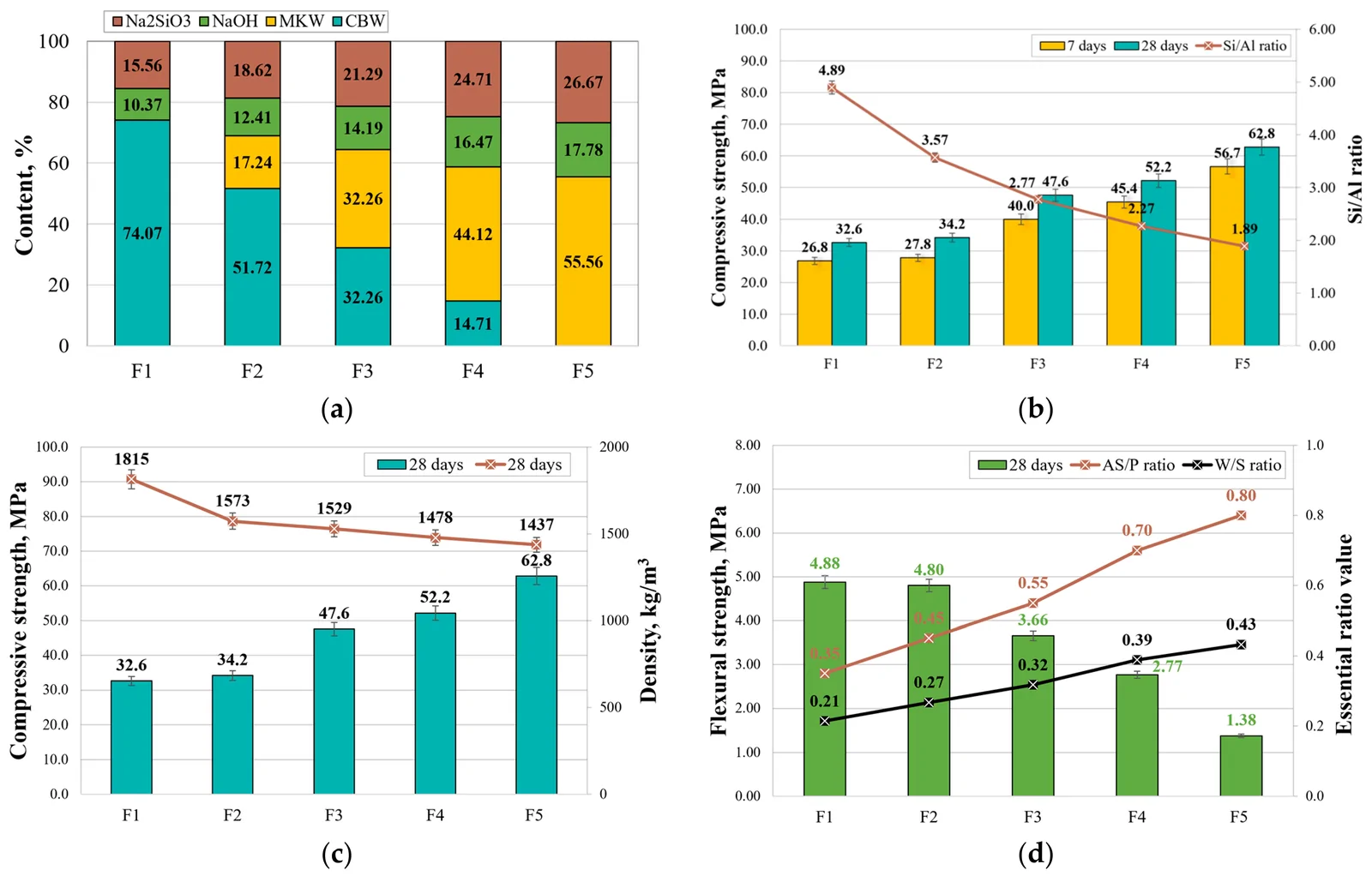
Mechanical properties of geopolymer binder: (**a**)—composition proportions; (**b**)—relationship between compressive strength at 7 and 28 days and Si/Al ratio; (**c**)—relationship between compressive strength and density at 28 days; (**d**)—relationship between flexural strength, AS/P and W/S ratio.

The compressive and flexural strength of geopolymers is pivotal in evaluating their mechanical performance and durability, rendering them suitable for structural and construction applications. The geopolymer binder’s mechanical properties, including compressive strength, flexural strength, and other essential strength relationships with important variables, are presented in Figure 7. The present study examined the development of geopolymer binder by utilizing two gradually combined aluminosilicate precursors: ceramic brick and metakaolin waste. The mechanical integrity of a geopolymer is contingent upon the composition of the oxide precursors utilized in its formulation. Ceramic brick waste, characterized by its lower reactivity (9.34% amorphous), was progressively substituted (25, 50, 75, 100 wt.%) with metakaolin waste, which exhibited a notably higher reactivity (73.07% amorphous). This substitution was implemented to ensure the incorporation of substantial amounts of amorphous Si and Al compounds into the designed geopolymer binders. The selected precursors were subjected to alkali activation with varying alkaline solution-to-precursor (AS/P) ratios, employing Na_2_SiO_3_ and NaOH activators at a constant ratio of 1.5 to ensure similar precursor activation processes and fresh mixture consistency, as delineated in compositions F1–F5 (see Figure 7a).

The geopolymer binder’s compressive strength at varying mixture design configurations after 7 and 28 days, along with its Si/Al ratio, is presented in Figure 7b. The reference specimens (F1) were composed of 100% CBW, and their compressive strength was found to be 26.8 and 32.6 MPa after 7 and 28 days, respectively. Silva et al. [59] report analogous results, utilizing solely CBW-based geopolymer with comparable activators and curing conditions. Also, the compressive strength can be enhanced through the optimization of the NaOH concentration, the water-to-binder ratio, and the SiO_2_/Na_2_O ratios. This study’s findings indicate that increased metakaolin waste content exerts a favorable influence on compressive strength, manifesting in a gradual enhancement. Notably, the MKW-derived geopolymer binder (F5) exhibited the highest compressive strengths of 56.7 and 62.8 MPa after 7 and 28 days, respectively. The highest compressive strength has been shown to be related to the proper selection of a Si/Al ratio of 1.89, resulting in a more cross-linked, stronger three-dimensional silicate network with improved geopolymer binder microstructure [60]. Duxson et al. [61] concluded that the Si/Al ratio plays a significant role in the mechanical behavior of metakaolin-based geopolymer. The optimum ratios in the range of 1.65–1.90 were identified as the most effective in achieving the highest compressive strengths, irrespective of the type of activator employed (sodium or potassium). Subsequently, Lahoti et al. [62] confirmed that geopolymer compressive strength increases with increasing Si/Al ratio, reaching a peak at a ratio close to 2.0. In addition, they demonstrated that compressive strength decreases with further increases in the Si/Al ratio. This is due to an excess of unreacted precursor particles, which act as defect sites and cause weakened geopolymer mechanical properties. In this instance, the ceramic brick waste precursor had lower reactivity, and an increased quantity of CBW resulted in a higher quantity of unreacted particles within the compositions. Consequently, compositions incorporating CBW (F1–F4) precursors exhibited diminished 28-day compressive strength when compared to the designed MKW-dominant compositions. In general terms, the incorporation of a Na_2_SiO_3_ activator and the use of a constant concentration of NaOH resulted in increased compressive strength in all the designed geopolymer binder compositions, when compared to our previous study [63], in which varying concentrations of NaOH were used as the activator alone. Despite the enhancement in strength resulting from the incorporation of Na_2_SiO_3_, signs of white plaque (efflorescence) were observed in compositions (F1) containing high contents of unreacted particles. It is therefore recommended that the optimum concentration of NaOH be selected to prevent the excess NaOH that may cause the formation of carbonate slats resulting from atmospheric carbonation [64].

The geopolymer binder’s density at varying mixture design configurations after 28 days, along with its compressive strength, is presented in Figure 7c. The hardened geopolymer binder density values exhibited a range from 1437 to 1815 kg/m^3^. It was established that as the content of CBW precursor within the designed compositions increases, so does the hardened geopolymer binder density. This phenomenon can be attributed to the higher particle density exhibited by the CBW precursor in comparison with the MKW precursor. Moreover, a parallel can be drawn with the conclusions of the preceding study [63]. The findings indicate that the density of geopolymer does not necessarily correlate with its compressive strength. This is evident in the present case due to the high content of CBW, which comprises a significant proportion of low-reactivity particles. These particles do not engage in the initial phase of geopolymerization, thus hindering the enhancement of compressive strength.

The flexural strength of the geopolymer binder at varying mixture design configurations after 28 days, along with its alkaline solution-to-precursor (AS/P) ratio and water-to-solids (W/S) ratio, is presented in Figure 7d. It was predicted that the flexural strength would exhibit a parallel trend with the compressive strength, and that the flexural strength would increase in proportion to the quantity of metakaolin waste precursor. Contrary results have been obtained, as the rise in metakaolin waste content led to a decrease in flexural strength, with the lowest recorded strength of 1.38 MPa occurring after 28 days in the composition (F5) containing 100% metakaolin waste precursor. The occurrence of this contradictory phenomenon can be attributed to the metakaolin-based geopolymer binder’s high open porosity, glassy structure and its notably high degree of brittleness. As previously discussed, to ensure similar geopolymer paste consistency (flowability) across the designed compositions, the addition of water was not an acceptable modification; rather, the alkaline solution content was increased with the gradual incorporation of MKW precursor, resulting in a higher alkaline solution-to-precursor (AS/P) ratio. The increase in the AS/P ratio led not only to higher alkaline content, but also to the appearance of excess water in the fresh geopolymer paste, as evidenced by the water-to-solids (W/S) ratio. The presence of excess water led to the formation of undesirable open porosity within the microstructure of the material. The high geopolymer binder open porosity is attributable to its capacity for water absorption.. In comparison, the F5 composition demonstrated a water absorption of 22.7% at the 28-day curing stage following a 24 h immersion phase. In contrast, only 9.7% absorption was observed in the F1 composition. Therefore, specimens composed exclusively of MKW precursor (F5) exhibited elevated open porosity, which resulted in diminished mechanical properties, particularly flexural strength. Additionally, a higher content of alkalis, especially an increased content of Na_2_SiO_3_, led to the formation of a high-alkaline geopolymer gel. This gave the hardened geopolymer a glassy matrix and a brittle structure. Along with high open porosity, this ultimately allowed it to perform optimally under compression but weakly under tension. Finally, the study results showed that the highest flexural strength of 4.88 MPa was obtained in the composition containing 100% ceramic brick waste (F1) precursor with the lowest AS/P and W/S ratios.

#### 3.2.2. Drying Shrinkage

**Figure 8:**
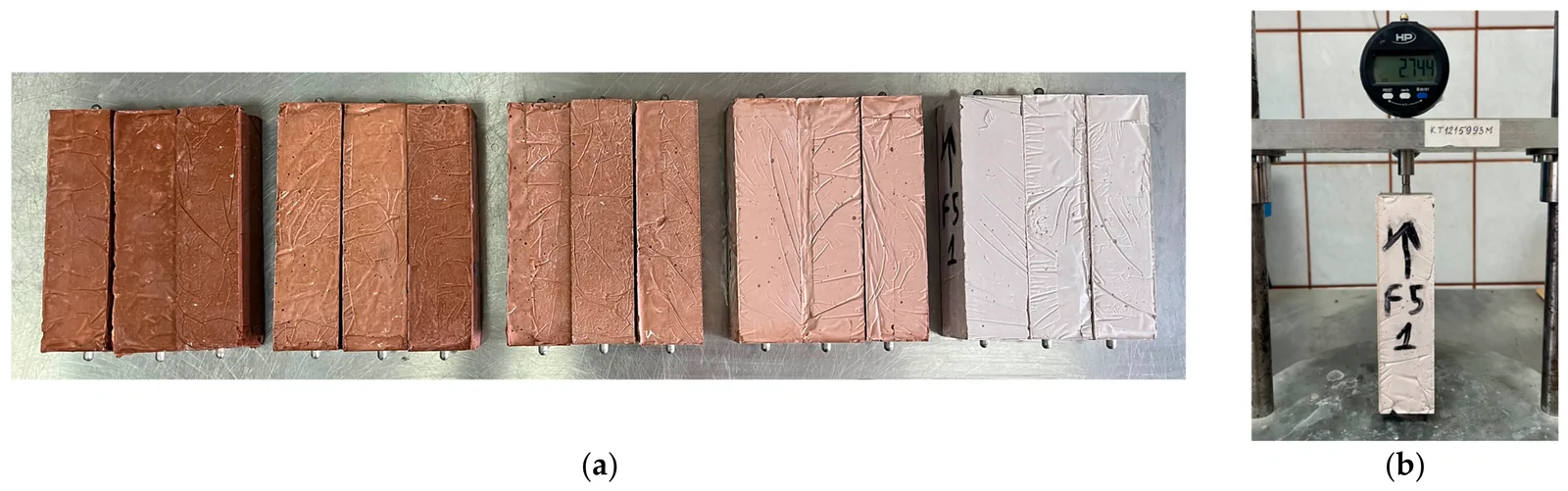
Geopolymer drying shrinkage test: (**a**)—binder prism specimens (F1–F5); (**b**)—length change measurement.

Shrinkage behavior of geopolymer binders is a critical factor in evaluating dimensional stability and long-term durability, thereby determining their suitability for structural and construction applications. The geopolymer binder specimens used for the drying shrinkage test are shown in Figure 8a, while the specimen length measuring procedure is illustrated in Figure 8b.

**Figure 9:**
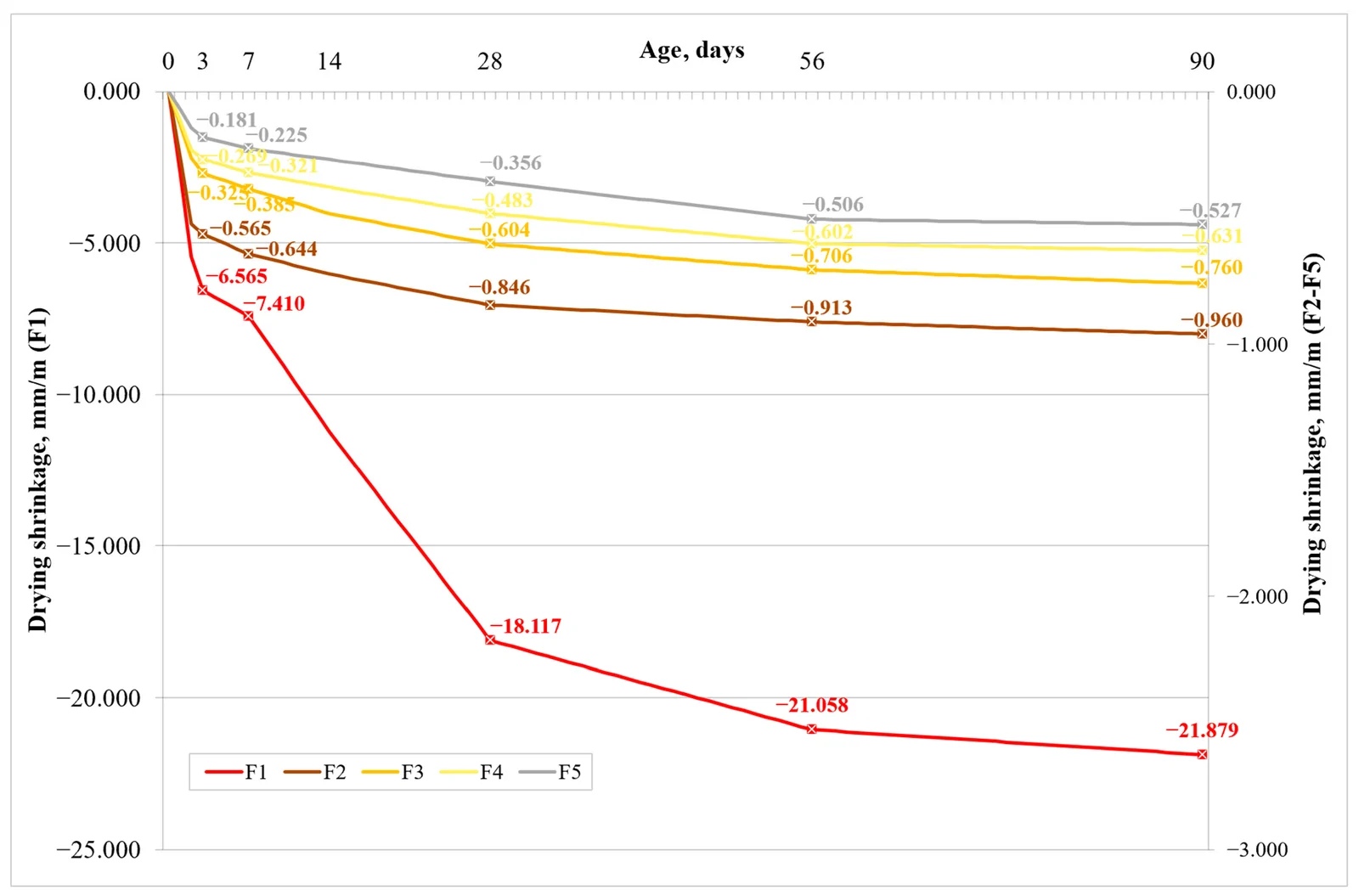
Geopolymer binder free-drying shrinkage after 90 days.

The geopolymer binder free-drying shrinkage measurement results are illustrated in Figure 9. It was observed that the majority of shrinkage occurs in the early stage of the curing process. A comprehensive analysis of geopolymer compositions reveals that an average of 41% of total 90-day contractions occurs within the first 3 days, and 78% total contractions occurring after 28 days of curing. Meanwhile, the remaining period of shrinkage (29 to 90 days) exhibited negligible contractions. The rapid and substantial shrinkage observed over the course of 3 to 28 days is attributable to two distinct mechanisms: capillary stress and disjoining pressure [65].

The reference specimens (F1), composed of 100% CBW, exhibited an exceptionally high 90-day drying shrinkage of 21.879 mm/m, accompanied by surface cracks. In contrast, compositions (F2, F3, F4, and F5) with increasing amounts of MKW precursor exhibited significantly lower drying shrinkages of 0.960, 0.760, 0.631, and 0.527 mm/m, respectively. It has been observed that a relatively modest quantity of metakaolin waste (25%), when incorporated as in composition F2, can substantially mitigate the shrinkage of geopolymer solely made of ceramic brick waste. This pronounced difference between F1 and the MKW-containing mixtures arises from fundamental differences in reaction degree, pore structure, and the form in which water is stored within the hardened matrix. The 100% CBW system (F1) contains a large fraction of unreacted, low-reactivity particles, which leads to a coarse and poorly connected pore network that retains substantial amounts of free water in larger capillaries. During drying, the retreat of water menisci in these wider pores generates high capillary tension, directly causing the large macroscopic shrinkage observed. A high Si/Al ratio of 4.89 indicates that ceramic brick waste precursors exhibit minimal dissolution in alkaline solutions due to their comparatively lower Al contents relative to their Si contents. This results in a diminished geopolymerization reaction rate, leading to the formation of an unstable aluminosilicate network [66]. The lowest Na/Al ratio of 0.68 indicates an absence of sodium, which is necessary to regulate the balance of the AlO_4_ tetrahedra, resulting in incomplete gel formation. Finally, even though the F1 composition exhibited the lowest W/S ratio of 0.21, it demonstrated the highest H_2_O/Na_2_O ratio of 15.45. The presence of an elevated H_2_O/Na_2_O ratio is indicative of a diluted alkaline environment with excess water. This excess water in F1 remains largely unbound and resides in capillary pores, making it highly susceptible to evaporation and generating substantial capillary stresses during drying. In contrast, the incorporation of MKW in F2–F5 produces a denser and more reactive geopolymer gel, which refines the pore structure, increases the proportion of chemically bound water, and significantly reduces the magnitude of capillary tension. As a result, MKW-rich mixtures exhibit far lower shrinkage despite containing higher total water contents. Therefore, the incorporation of metakaolin waste in the geopolymer binder system results in an intensified geopolymerization reaction due to high MKW particle reactivity. Consequently, it facilitates the regulation of the ceramic brick-based geopolymer binder, which is susceptible to significant shrinkage.

Studies on the drying shrinkage of solely activated ceramic brick precursor geopolymers are currently scarce. As indicated by the findings of Migunthanna [67], the incorporation of modest quantities of CBW precursor into geopolymer systems has been observed to result in increased drying shrinkage under unsealed curing conditions.

Furthermore, shrinkage of geopolymer binders may be mitigated by adjusting the CBW/MKW precursor composition and particle fineness, as well as by incorporating suitable aggregates that can restrain matrix deformation. Sealed curing has also been demonstrated to limit moisture loss and reduce drying shrinkage. It is notable that these approaches may prove particularly advantageous in the context of CBW-rich compositions, which are characterized by pronounced shrinkage.

**Figure 10:**
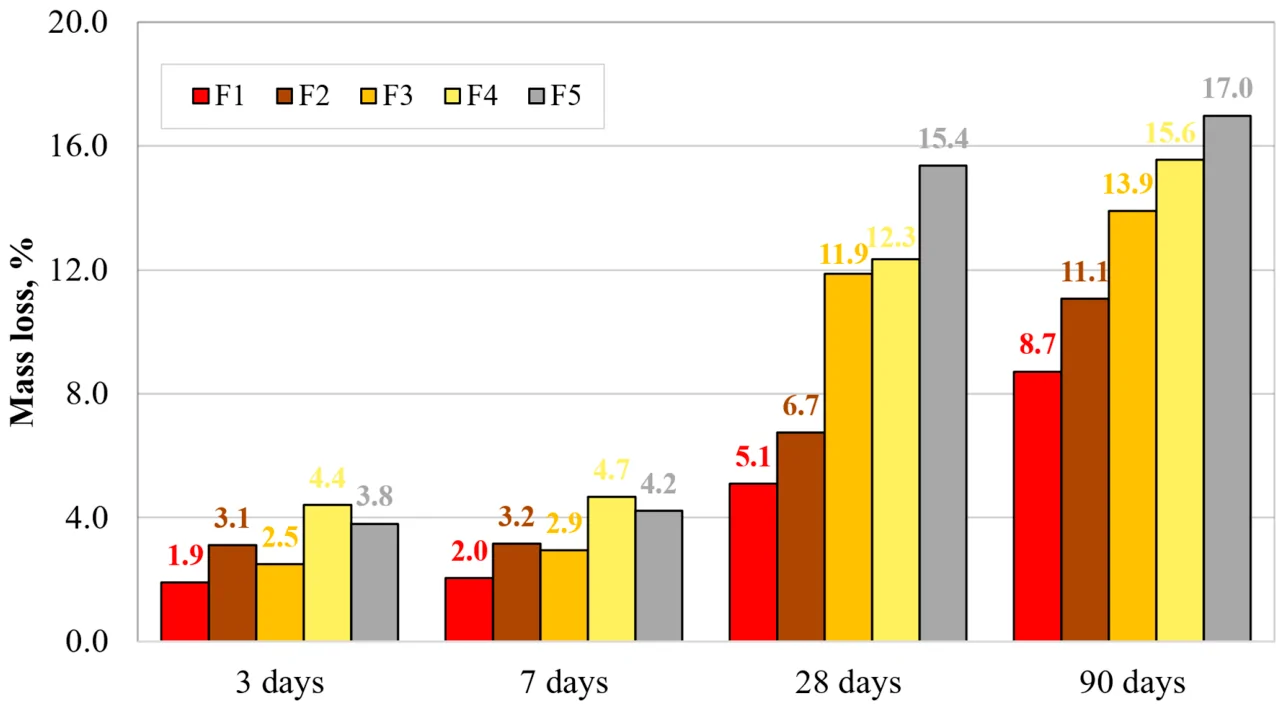
Change in mass throughout drying shrinkage.

During the execution of the free-drying shrinkage evaluation, a discernible alteration in the mass of concrete specimens was observed, as illustrated in Figure 10. The mass change after 90 days for the F1 reference composition specimens was the lowest (8.7%), while the compositions of F2–F5, which contained increasing amounts of the metakaolin precursor, led to an increase in mass loss (11.1–17.0%). This is a contradictory result, as the geopolymer binder compositions (F2–F5) with lower drying shrinkage values achieve the highest mass changes.

This apparent contradiction is resolved by recognizing that mass loss reflects the total amount of evaporable water, whereas shrinkage reflects the mechanical response of the pore network to water removal. MKW-rich mixtures contain more total water due to higher AS/P and W/S ratios, and their finer, more connected pore networks allow this water to evaporate more readily, resulting in greater mass loss. However, because much of this water is stored in finer pores or is partially bound within the geopolymer gel, its removal generates far lower capillary stresses, leading to minimal shrinkage. According to Yang et al. [68], the mass loss trend of cement-based systems is more consistent with increased shrinkage. This is because metakaolin-based geopolymer systems lose significantly more mass due to the presence of additional free water, and because the geopolymerization reaction uses less water than the ordinary cement hydration reaction. Additionally, Ling et al. [69] state that geopolymer mass loss depends heavily on the curing conditions, especially relative humidity, with mass loss increasing sharply at RH levels of 60%, although this does not guarantee high drying shrinkage.

### 3.3. Durability Properties

#### 3.3.1. Macroscopic Evaluation After Thermal Exposure

**Figure 11:**
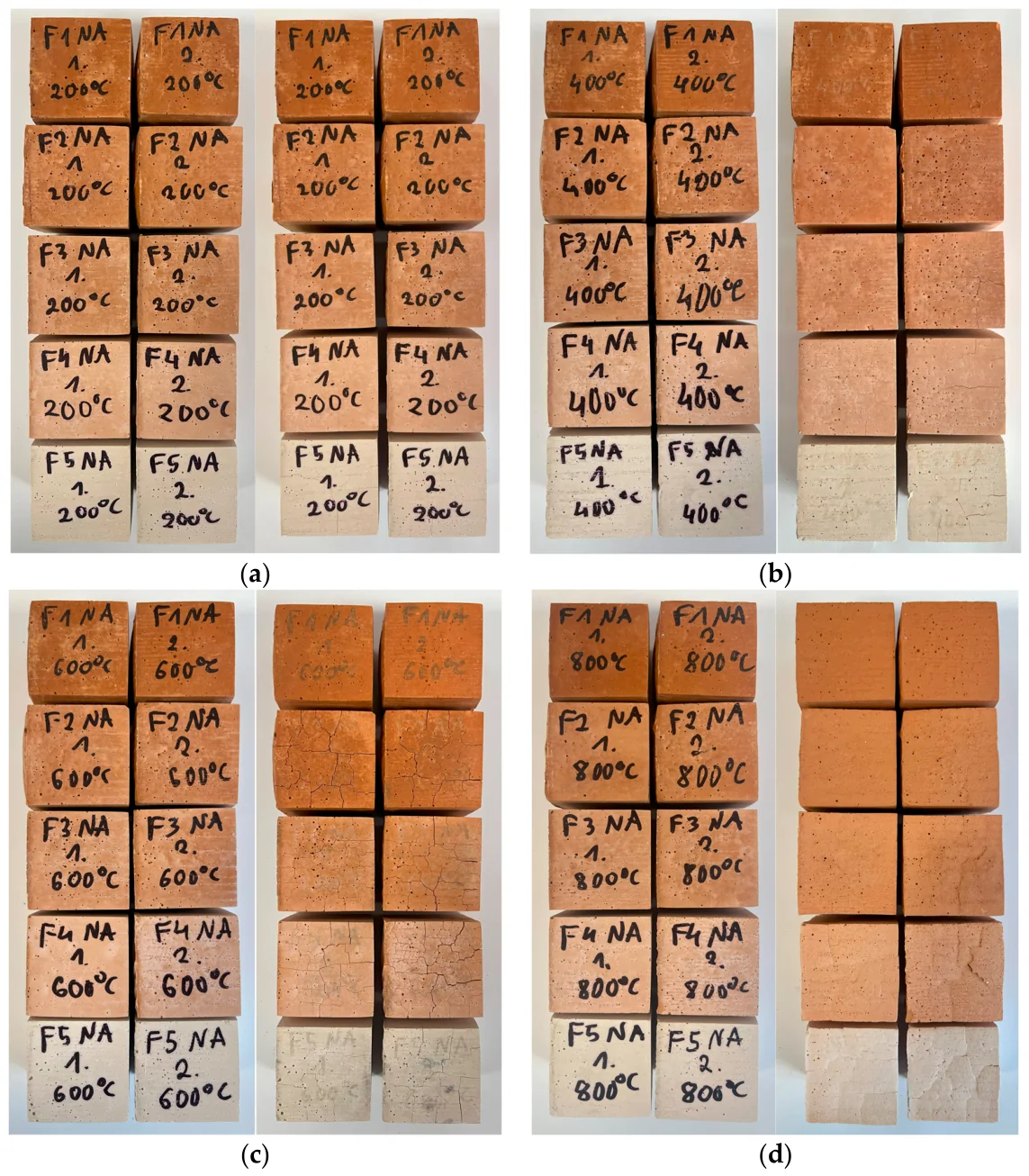
Macroscopic images of geopolymer binder specimens before and after elevated temperature exposure: (**a**)—200 °C; (**b**)—400 °C; (**c**)—600 °C; (**d**)—800 °C.

Macroscopic evaluations were conducted post-thermal exposure after 200, 400, 600 and 800 °C temperature treatment to appraise visible changes in the geopolymer binder specimens. These included surface degradation, discoloration, cracking, and other structural alterations that are indicative of thermal instability. The geopolymer binder specimens used for visual inspection before and after a series of temperature treatments are illustrated in Figure 11.

The initial observable change pertains to the color of the geopolymer binder at elevated temperature exposure. It has been observed that with an increase in temperature, geopolymer binder compositions containing at least minor amount of ceramic brick precursors are prone to a loss of color intensity. This phenomenon can be attributed to the oxidation of iron compounds at elevated temperatures [70], with ceramic brick waste precursor containing approximately 5% Fe_2_O_3_. The other salient visual alteration is the occurrence of cracking. The most crack-prone geopolymer binder compositions were found to be F5 and F4, which exhibited the highest W/S ratios, as initial cracking started at a temperature exposure of 200 °C (see Figure 11a). It was demonstrated that compositions F3 and F2 exhibited slightly enhanced cracking performance, with the formation of surface cracks occurring at 600 °C (see Figure 11c). The composition that exhibited the highest degree of resistance to cracking was F1, which was composed exclusively of ceramic brick waste precursor, with minor cracking observed at 800 °C (see Figure 11d). The underlying cause of cracking is the presence of excess water (high W/S ratio), which does not contribute to the geopolymerization reaction. This results in elevated geopolymer binder open porosity, thereby facilitating rapid water evaporation from the specimen matrix through the capillary pores [71].

#### 3.3.2. Thermal Shrinkage

**Figure 12:**
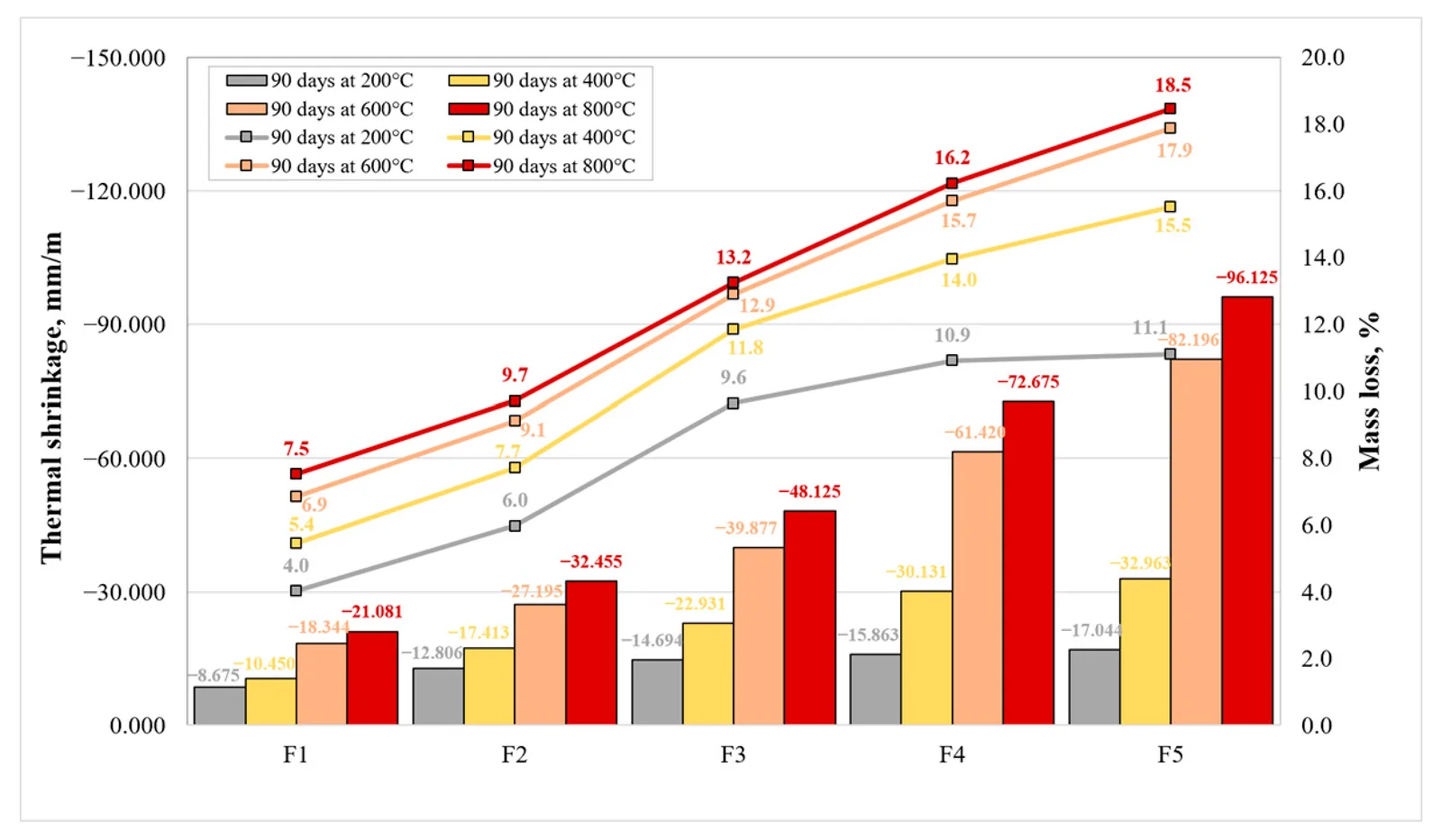
Relationship between thermal shrinkage and mass loss of the geopolymer binder after 90 days.

Thermal shrinkage is a critical factor in determining the dimensional stability of geopolymer binders, with substantial consequences for their residual mechanical properties. The thermal shrinkage of the geopolymer binder specimens was evaluated through the implementation of the constant heating rate method at temperatures of 200, 400, 600, and 800 °C, with a heating rate of 5 °C/min for a duration of 2 h (see Figure 4). The relationship between geopolymer binder thermal shrinkage and mass loss is illustrated in Figure 12.

**Figure 13:**
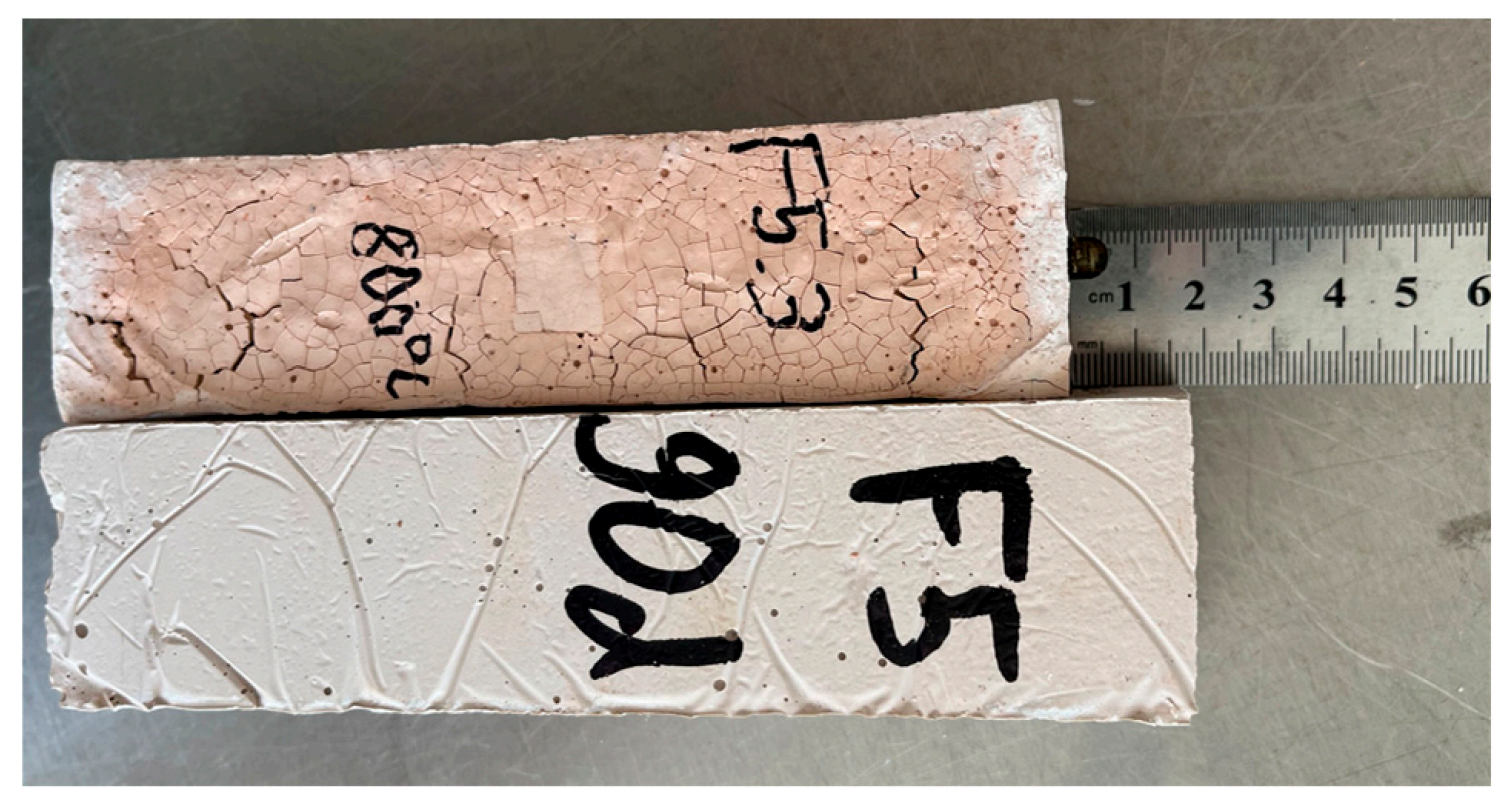
Visualization of extreme thermal shrinkage at 800 °C in composition F5.

It was found that the composition (F1), comprising solely ceramic brick waste precursor, yielded the most efficacious performance, characterized by the lowest thermal shrinkage. The material was demonstrated to exhibit the lowest levels of thermal shrinkage and mass loss across the range of selected temperatures. The highest values were recorded at 800 °C, with thermal shrinkage measuring 21.081 mm/m and mass loss amounting to 7.5%, accompanied by an absence of cracking or spalling effects that are attributed to the loss of free water and subsequent condensation of hydroxyl groups [72]. Furthermore, an observation was made that the F1 composition exhibits the highest Si/Al ratio of 4.89 across the designed geopolymer binder compositions. According to Thokchom et al. [73], this ratio correlates with enhanced thermal stability. The hypothesis was formulated that the extent of geopolymer binder mass loss would directly correspond to the degree of binder thermal shrinkage. Furthermore, an increase in MKW precursor content in geopolymer binder results in greater mass loss and thermal shrinkage. It was established through visual observation that the F5 compositions underwent the greatest mass loss and thermal shrinkage at each of the elevated temperatures that were selected for analysis. At an elevated temperature of 800 °C, the metakaolin waste-based geopolymer binder composition (F5) exhibited a substantial decrease in size, measuring 96.125 mm/m, accompanied by a notable mass reduction of 18.5%. This phenomenon resulted in substantial cracking, as illustrated in Figure 13, and a significant decline in residual mechanical properties.

The study results confirm a statement by Salahuddin [74], that the most rapid geopolymer thermal shrinkage is observed under temperatures ranging from 650 to 800 °C.

#### 3.3.3. Residual Compressive and Flexural Strength

**Figure 14:**
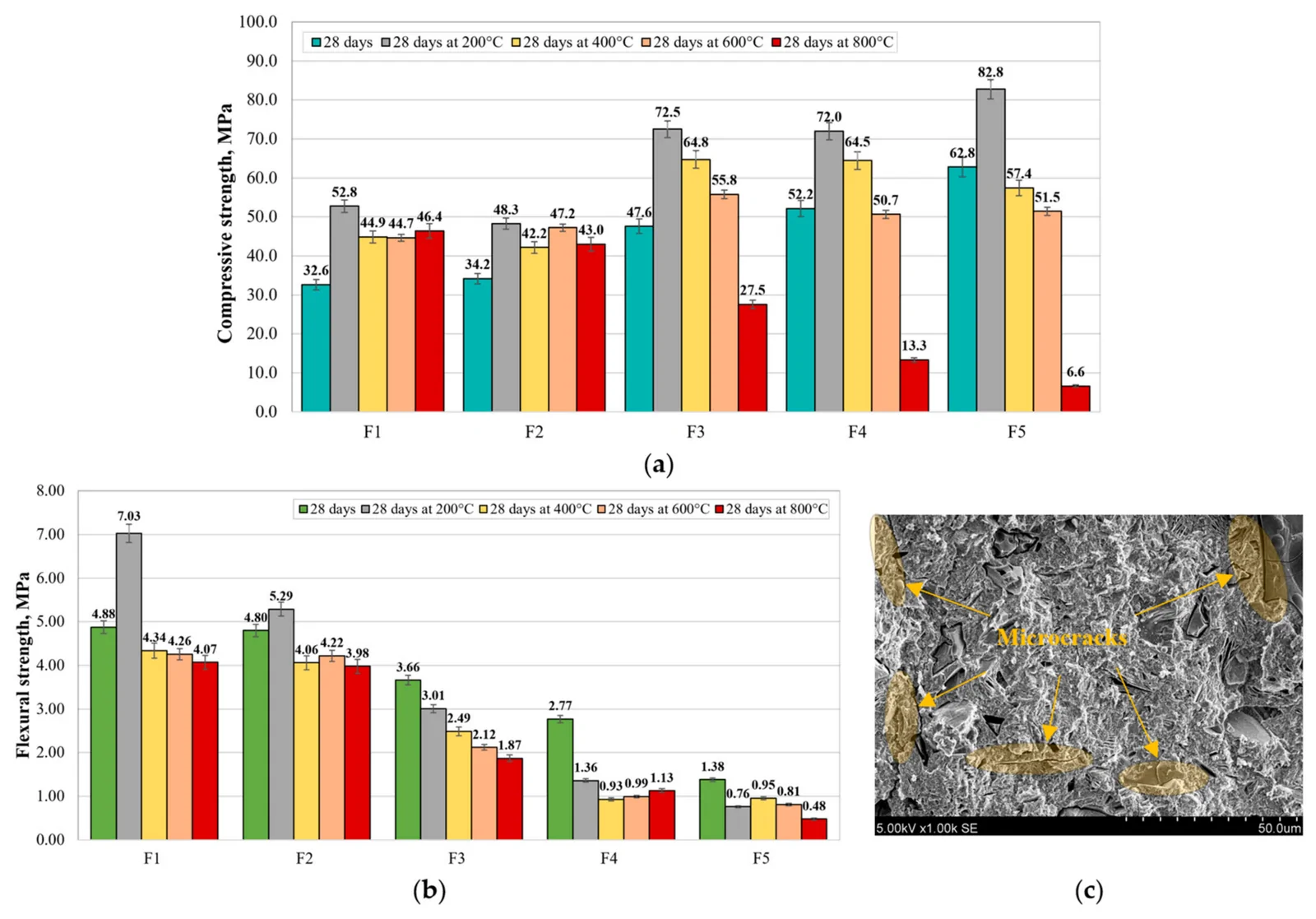
Mechanical properties of geopolymer binder after elevated temperature exposure: (**a**)—compressive strength; (**b**)—flexural strength, (**c**)—microcracks in composition F5 at 400 °C.

A comprehensive evaluation of the residual mechanical properties of geopolymer binders under conditions of high-temperature exposure is imperative to ascertain their viability for utilization in fire-resistant structures and thermally demanding applications, wherein post-fire integrity is of paramount importance. The geopolymer binder’s compressive strength at a range of elevated temperatures and varying mixture design configurations following 28 days is presented in Figure 14a, while the flexural strength is presented in Figure 14b, along with a SEM image of crack formation in the MKW-based composition (F5) at 400 °C in Figure 14c.

The subsequent compressive and flexural strength analysis involved a comparison of the strength outcomes both prior to and following exposure to elevated temperatures. The experimental data demonstrate that geopolymer binder exposure to 200 °C resulted in a marked enhancement in compressive strength across all the compositions under investigation (F1: 61.8%, F2: 41.2%, F3: 52.3%, F4: 38.0%, F5: 31.8% increase compared to no thermal exposure), with composition F5 demonstrating the highest compressive strength of 82.8 MPa. The observed increase in strength at 200 °C is attributed to secondary geopolymerization and further polycondensation reactions. These processes have been demonstrated to enhance interparticle bonding, promote matrix densification, and improve the microstructure of the geopolymer binder. It is notable that no sintering or crystallization occurs at this temperature; however, the thermal curing process does promote the formation of a more compact and mechanically robust amorphous network [75,76].

The findings indicate that geopolymer binder exposure to 400 °C resulted in a slight decrease in strength when compared with results after 200 °C exposure. However, an enhancement in compressive strength was observed across the F1–F4 compositions when compared to no thermal exposure (F1: 37.5%, F2: 23.4%, F3: 36.1%, F4: 23.6% increase). The composition F3 demonstrated the highest compressive strength of 64.8 MPa at 400 °C. The compositions that were composed entirely of MKW precursor demonstrated a slight diminution (F5: 8.6%) in compressive strength at 400 °C. Meanwhile, analogous outcomes were attained at 600 °C, with a conspicuous augmentation in strength exhibited by the F1–F3 compositions, in contrast to the absence of thermal exposure (F1: 36.9%, F2: 38.2%, F3: 17.2%). The composition F3 was observed to yield the most significant strength, with a value of 55.8 MPa. The compositions F4–F5, with higher MKW content, exhibited a slight decrease in compressive strength at 600 °C (F4: 2.9%, F5: 18.1%). Consequently, the compressive strength of the geopolymer binder matrix demonstrated stability within the range of 400 to 600 °C, with a slight decrease observed. This finding indicates adequate thermal stability within this temperature range, suggesting that the geopolymer binder matrix maintains its structural integrity and resistance to thermal degradation at these temperatures.

The findings of the compressive strength tests on geopolymer binders subjected to 800 °C of high-temperature exposure revealed notable variations in the strength retention of different compositions. A substantial decline in strength was observed in binder compositions containing 50% or more metakaolin waste precursor (F3: 42.1, F4: 74.5%, F5: 89.5%), while compositions containing 75% or more ceramic brick waste precursor demonstrated resilience and retained their initial compressive strength with a modest augmentation (F1: 42.3%, F2: 25.8%). The sintering effect is comprehensively delineated in the study by Pan et al. [77]. The study demonstrates that the sintering effect can either augment strength through further geopolymerization and enhanced ductility or diminish strength due to thermal incompatibility, which manifests as increased brittleness. In this instance, the metakaolin-based geopolymer binder demonstrated a reduction in strength, attributable to thermal incompatibility. It is also crucial to note that the ceramic brick waste precursor-based geopolymer binders (F1 and F2) exhibited a high Si/Al ratio (4.89 and 3.57), which, in accordance with the study by Lahoti et al. [78], enhances thermal stability. Furthermore, a high content of unreacted ceramic brick waste particles was found to be affected by sintering, which led to the subsequent geopolymerization process. This resulted in the strengthening and stabilization of the geopolymer binder through thermal means. The findings of this study demonstrate that geopolymers produced from ceramic brick waste possess the capacity to endure temperatures of more than 800 °C without significant strength loss.

The results demonstrate that the degradation in flexural strength is considerably more pronounced than that observed for compressive strength after exposure to elevated temperatures. This disparity is primarily attributed to the greater sensitivity of flexural strength to microstructural damage, including microcrack initiation, crack propagation, and pore evolution under thermal loading conditions [79]. A progressive reduction in flexural strength was recorded at 400, 600, and 800 °C, indicating a temperature-dependent deterioration mechanism. This decline is associated with increased alkaline solution-to-precursor (AS/P) and water-to-solid (W/S) ratios, in combination with the incorporation of metakaolin waste as a precursor, which collectively promoted the development of higher open porosity. Such porous structures exhibit limited thermal stability and reduced resistance to crack initiation. Scanning electron microscopy (SEM) analysis (Figure 14c) revealed the presence of microcracks at 400 °C; moreover, with further temperature elevation, these cracks became progressively wider and more interconnected, reflecting intensified thermal incompatibility and matrix degradation. The enlargement and coalescence of cracks at 600 and 800 °C significantly compromised structural integrity, thereby accelerating the loss of residual flexural strength. The only enhancement in flexural strength was observed at the 200 °C temperature exposure in geopolymer binder compositions composed primarily of ceramic brick waste (F1: 44.1%, F2: 10.2% increase). This enhancement can be attributed to the previously mentioned secondary geopolymerization and further polycondensation reactions.

#### 3.3.4. Water Resistance

**Figure 15:**
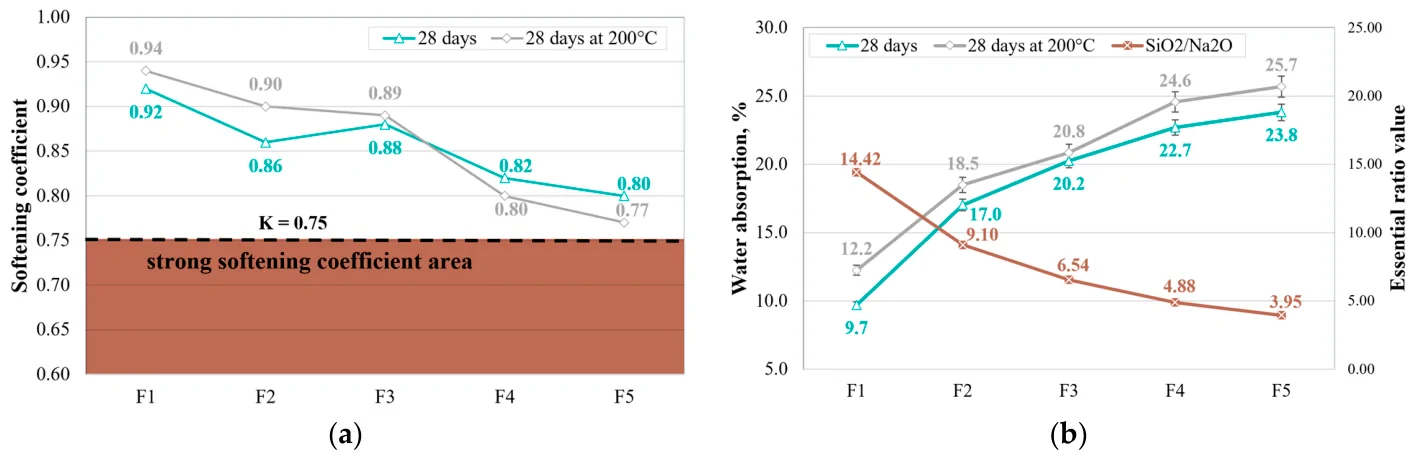
Water resistance of geopolymers after 28 days of curing and after thermal treatment at 200 °C: (**a**) softening coefficient; (**b**) water absorption.

The water resistance of geopolymer binders is described by two parameters: the softening coefficient and water absorption. These parameters are critical for evaluating geopolymer durability, as they provide insight into porosity, moisture ingress, and long-term performance. The water effects of the geopolymer binder were investigated by exposing specimens to hydration for a period of 24 h [80]. The softening coefficient and water absorption parameters were subjected to testing following a 28-day period of specimen curing and exposure to a temperature of 200 °C for a duration of 2 h, as illustrated in Figure 15.

The geopolymer binder softening values displayed in Figure 15a illustrate the impact of specimen soaking in water on compressive strength preservation, while the water absorption values presented in Figure 15b demonstrate the extent to which water penetrates the geopolymer binder matrix following immersion. The geopolymer binder is classified as a weakly softened material when its softening coefficient value (K) exceeds 0.75 [81]. It was observed that not a single geopolymer composition exhibited a softening coefficient value lower than 0.75, indicating that the designed binders demonstrate only minimal loss of strength when exposed to water. However, a discernible trend emerged from the data analysis: as the metakaolin waste precursor content increased, the softening coefficient of specimens aged 28 days decreased (F1: 0.92, F2: 0.86, F3: 0.88, F4: 0.80, F5: 0.77). The observed phenomenon can be attributed to an increase in open porosity, which is associated with a gradual increase in excess water within the geopolymer binder matrix. In essence, the increase in alkaline solutions was employed to enhance the geopolymerization process and comparable mixture consistency through all compositions. Concurrently, the alkaline solution-to-precursor (AS/P) ratios, along with the water-to-solids (W/S) ratios, underwent an increase. Consequently, an increase in W/S ratios resulted in elevated water absorption through the 28-day-aged geopolymer binders (F1: 9.7%, F2: 17.0%, F3: 20.2%, F4: 22.7%, F5: 23.8%). Furthermore, the findings of the present study corroborate the hypothesis that higher SiO_2_/Na_2_O ratios, lower Na_2_O content and lower excessive water content result in reduced water absorption and enhanced softening coefficient values (see Figure 15b). This conclusion is analogous to those reported by other researchers [82,83].

The water absorption values after 200 °C temperature exposure demonstrates a gradual increase, attributable to mass loss during the heating process. The softening coefficient values after 200 °C temperature exposure exhibited an enhancement to a certain extent (compositions F1, F2, F3), following a minor diminution (compositions F4, F5). This phenomenon can be attributed to the enhanced geopolymerization process, whereby unreacted ceramic brick waste precursor particles become involved in the reaction. This results in a reorganization of gel phases, reduction of porosity, the removal of free water, and the formation of a denser, stronger structure with reduced water sensitivity. Therefore, the optimal softening coefficient values were obtained in the composition that was entirely composed of CBW precursor, with a maximum value of 0.94 after 200 °C heat treatment. These values demonstrate enhancement in comparison to the findings reported in a study by Li et al. [84].

### 3.4. Microstructural and Phase Analysis

#### 3.4.1. XRD Analysis

**Figure 16:**
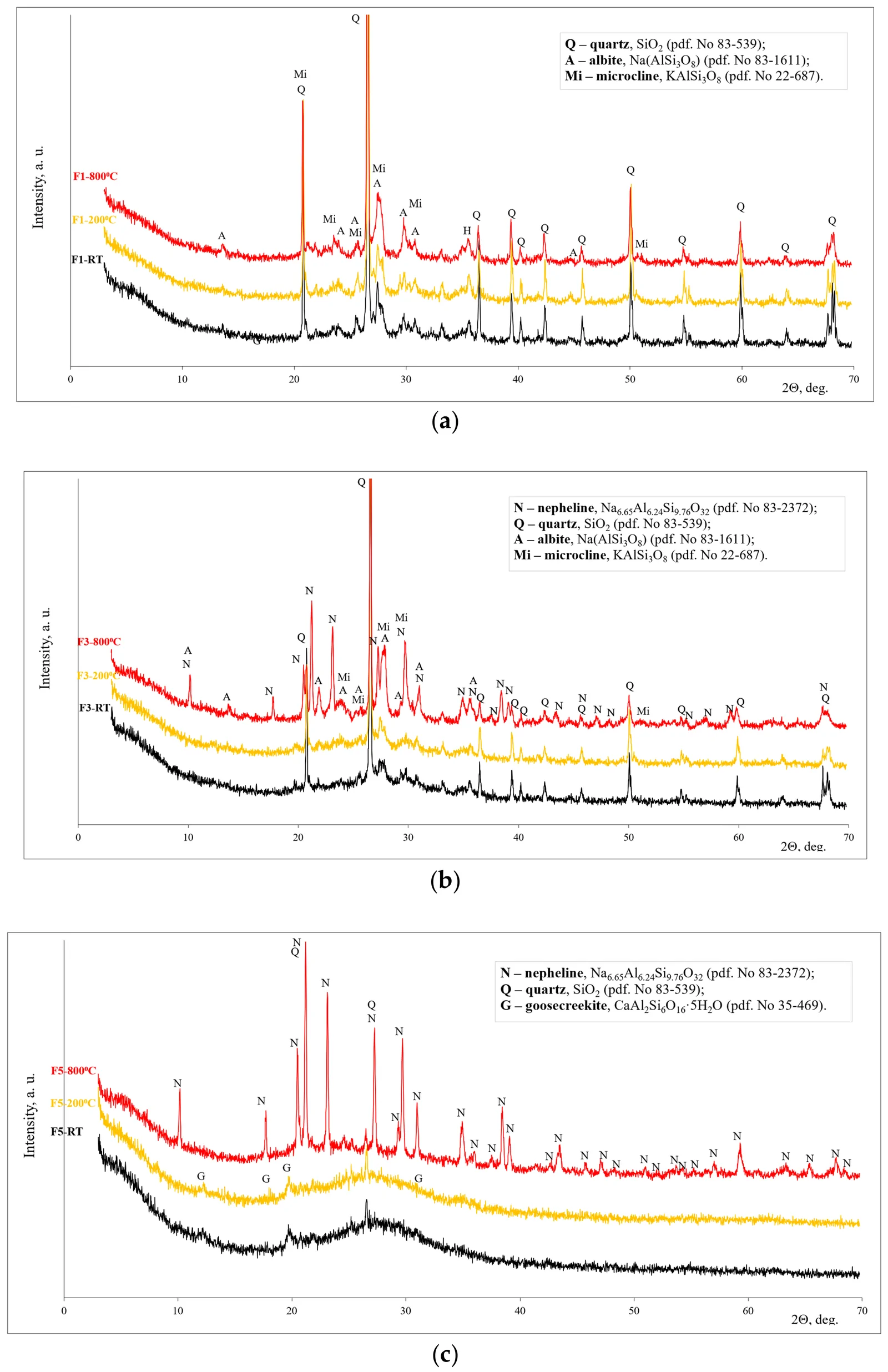
Mineralogical changes in geopolymer binders revealed by XRD analysis at room temperature, 200 °C, and 800 °C for compositions: (**a**)—F1, (**b**)—F3, (**c**)—F5.

The mineral compositions of three geopolymer binder compositions (F1, F3, and F5) were determined using XRD analysis, as illustrated by the mineralogy curves in Figure 16. The following geopolymer binder compositions were selected on the basis of the highest compressive strength values recorded both prior to and following high-temperature treatment (200 °C and 800 °C), with the understanding that elevated temperatures have been shown to exert a positive influence on the mechanical properties of geopolymer binders.

Following a 28-day geopolymerization process, quartz, microcline, and albite were identified in the F1 composition specimens as crystalline compounds (Figure 16a). These minerals constitute the predominant crystalline phases observed in ceramic brick waste precursor, aligning with the minerals identified by Mahmoodi et al. [85]. The F1 specimens (geopolymer binder composed of 100% CBW precursor) subjected to high temperatures exhibited mineral compositions analogous to those observed in the specimens prior to exposure (Figure 16a). Consequently, elevated temperatures do not induce alterations in the mineral composition of CBW-based geopolymer binders, thereby preserving their compressive strength.

The experiments demonstrated that when the geopolymer binder was prepared from a blend of CBW and MKW precursors (composition F3), no significant changes were observed after exposure to 200 °C (see Figure 16b). Nonetheless, nepheline mineral formation has been observed following exposure to temperatures of 800 °C. As Melinescu et al. [86] also demonstrated, nepheline may be synthesized from calcined kaolin and fly ash at a temperature of 800 °C for a duration of 2 h.

The geopolymerization of the MKW-based geopolymer binder (composition F5) was shown to produce a high proportion of amorphous compounds, with a negligible amount of goosecreekite as a crystalline compound (Figure 16c). The goosecreekite compounds were identified in a previous study [87]. Following exposure to a temperature of 200 °C, the mineral composition of the binders remained unaltered. However, at 800 °C, nepheline compounds became the predominant mineral composition.

#### 3.4.2. FT-IR Analysis

**Figure 17:**
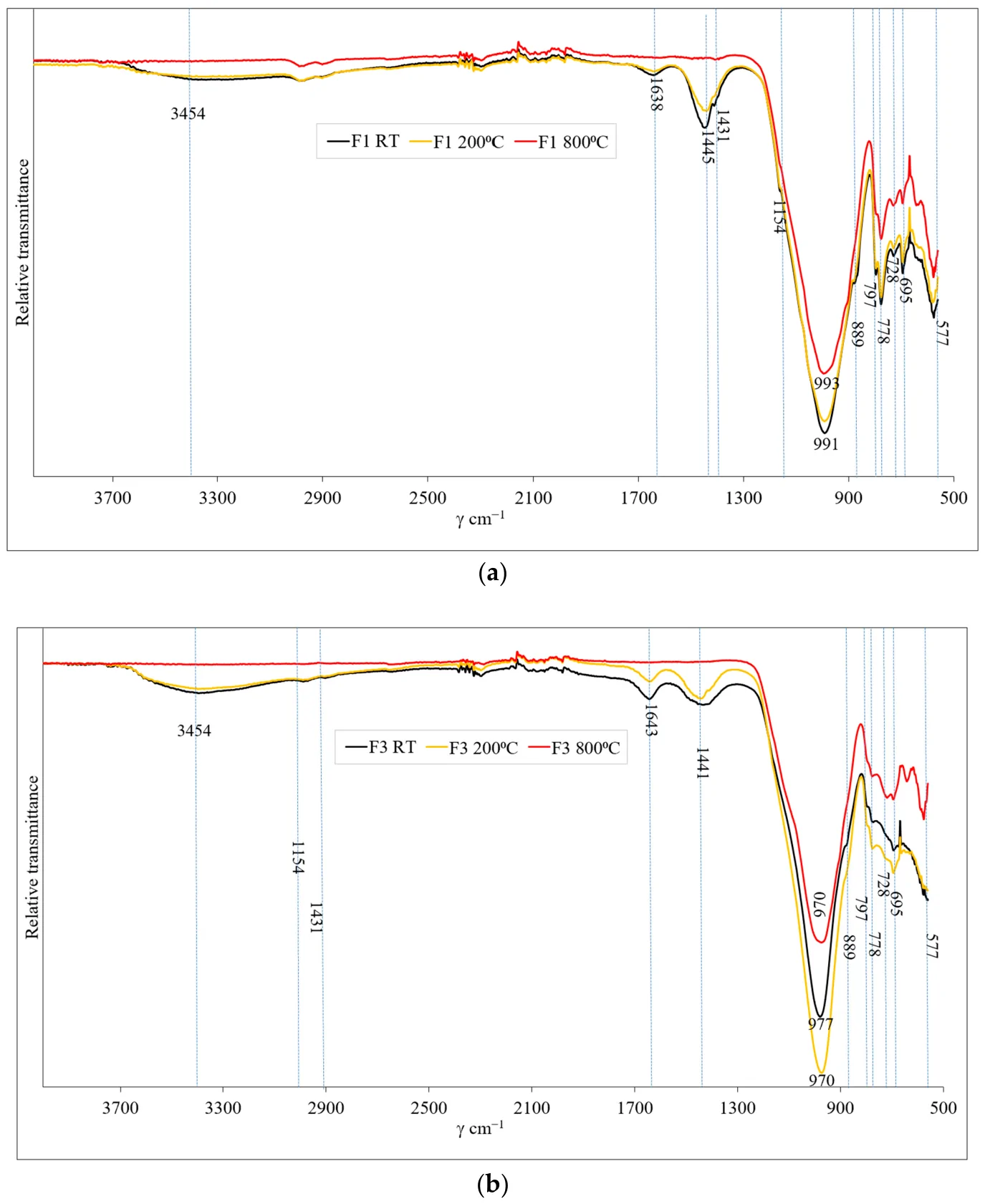

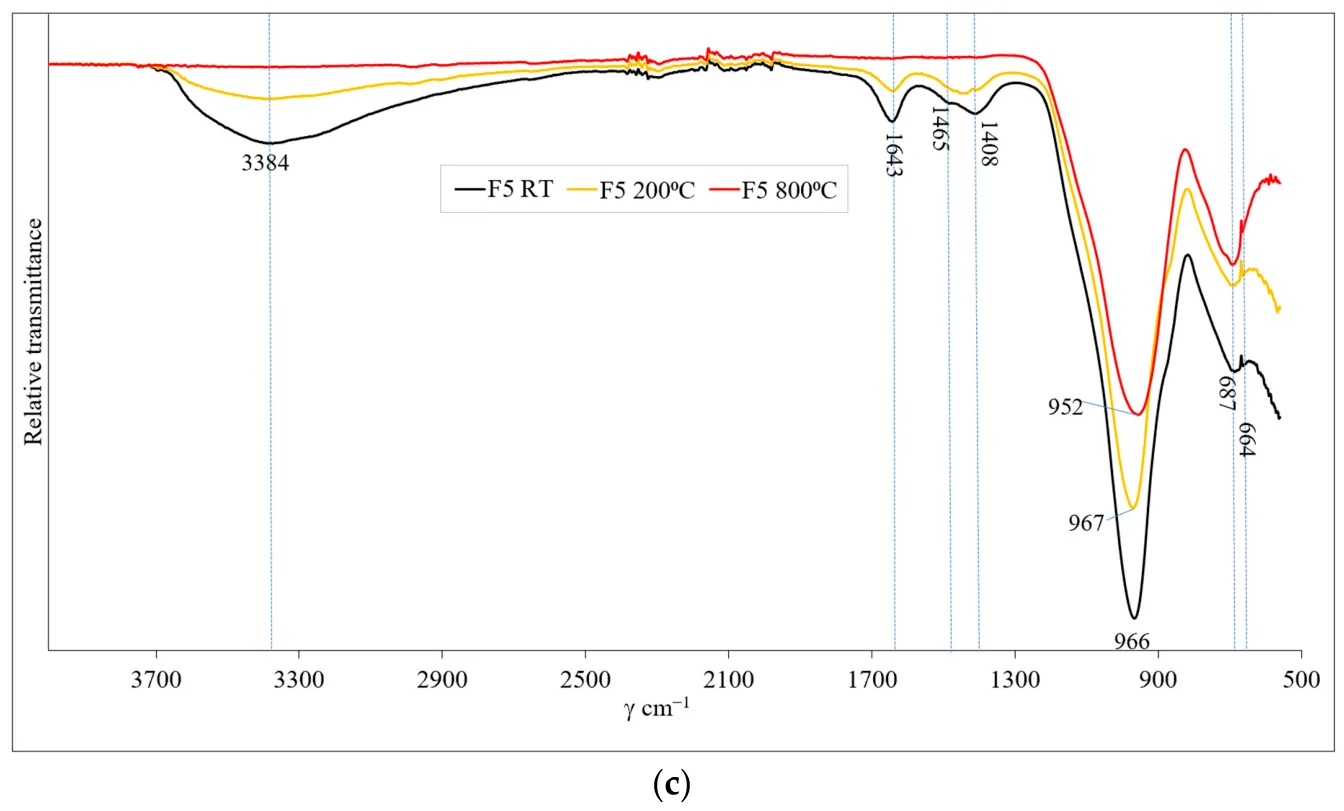
Mineralogical changes in geopolymer binders revealed by FT-IR analysis at room temperature, 200 °C, and 800 °C for compositions (**a**)—F1, (**b**)—F3, and (**c**)—F5.

The findings from the FT-IR analysis, performed on the same specimens used in the XRD analysis, served to verify the results of the XRD analysis and detected amorphous compounds. The FT-IR spectrum of geopolymer binders (F1) made from 100% CBW precursor is shown in Figure 17a. The bands around 3454 cm^−1^ (O–H stretching vibrations) and 1638 cm^−1^ (H–O–H bending vibrations) are typically associated with hydroxyl groups or adsorbed molecular water. When the exposure temperature was increased, the intensity of these bands decreased. The reduction of the 3454 cm^−1^ peak suggests thermal dehydration and possibly condensation of silanol groups, resulting in a more compact or crystalline structure. The decrease in the intensity of the 1638 cm^−1^ peak indicates that the material loses bound water, which is typical during geopolymer thermal treatment [88]. When MKW was introduced into the system (specimens F3 and F5), the intensity of the bands around 3454 cm^−1^, 1638 cm^−1^, and 1643 cm^−1^ gradually increased (Figure 17b,c), suggesting enhanced water retention or increased hydroxyl content due to the modified precursor composition. However, in all cases, when the specimens were exposed to 800 °C, these bands disappeared, indicating complete dehydration and the possible formation of a more crystalline or thermally stable structure such as nepheline.

The absorption band appearing at 1408 and 1465 cm^−1^ is attributed to carbonate stretching and can be attributed to CaCO_3_ and Na_2_CO_3_ [89]. After exposure to high temperatures, a decrease in the intensity of the bands was observed, and after treatment at 800 °C, it eventually disappeared.

Asymmetric stretching vibrations of Si–O, observed between 688–797 cm^−1^, 778 cm^−1^, and 1154 cm^−1^, are attributed to unreacted and/or partially reacted quartz [90]. After thermal treatment at 200 °C and 800 °C, the intensity of these bands decreased, indicating progressive transformation or consumption of quartz during the geopolymerization and heat exposure processes. Notably, quartz was detected only in specimens where the precursor was composed of CBW, suggesting that CBW contributes to the presence of crystalline silica phases in the initial material.

At 991 cm^−1^ and 993 cm^−1^, the most intense and broad bands were detected, which can be attributed to the presence of X-ray amorphous gel [90]. At 200 °C, the intensity of these bands slightly increased, likely due to geopolymerization reactions occurring within the gel structure (Figure 17a,c).

At 800 °C, a reduction in band intensity was observed, accompanied by a shift to higher wavenumbers, indicating structural changes such as dehydration, densification, and crystallization of the amorphous phase (Figure 17a). In contrast, for specimens whose precursor was made from MKW, this band shifted to lower wavenumbers, which may be attributed to the incorporation of aluminum into the gel structure, affecting the Si–O–Al network and altering the vibrational characteristics (Figure 17c).

#### 3.4.3. SEM Analysis

**Figure 18:**
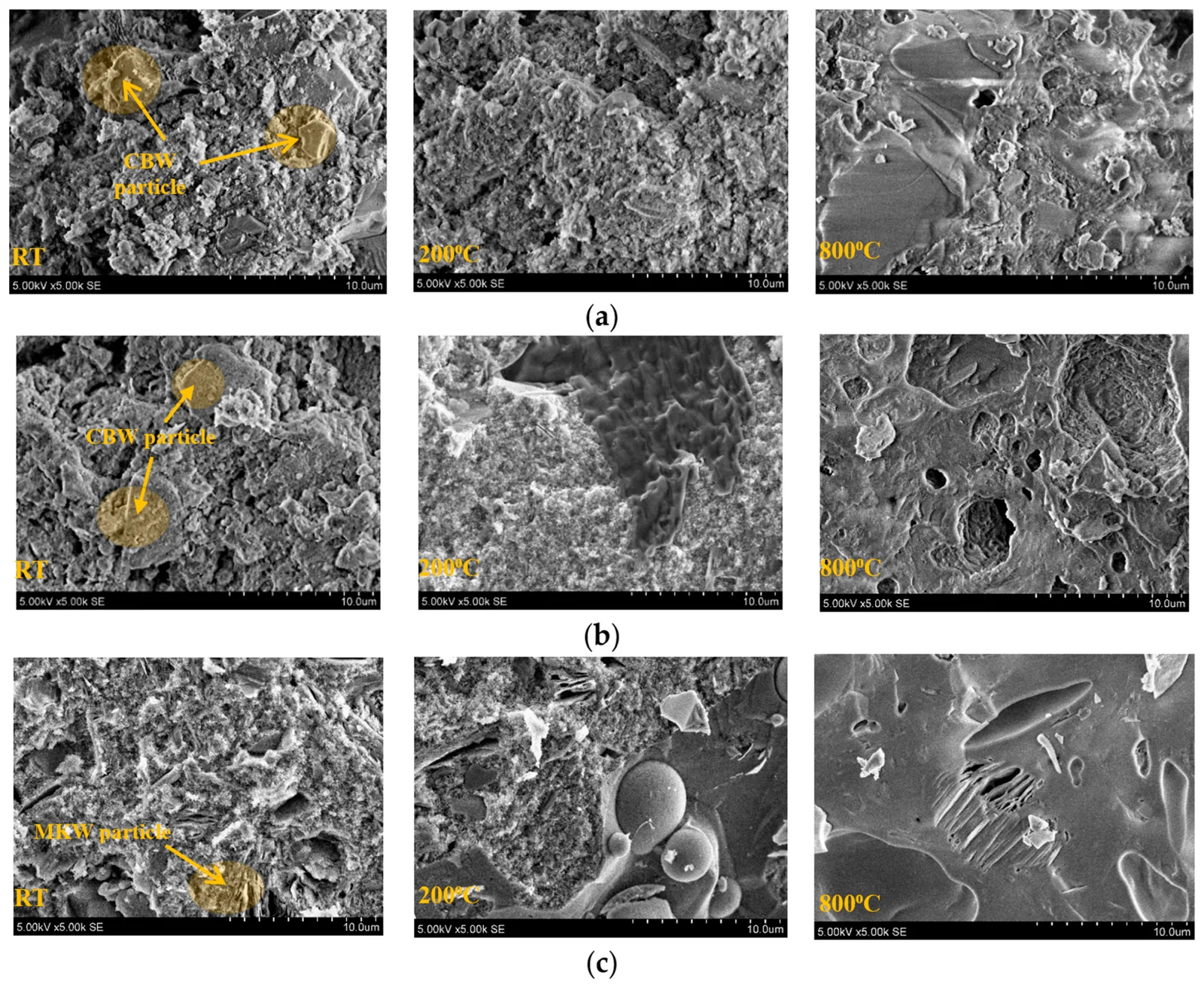
SEM images of geopolymer binder at room temperature, 200 °C, and 800 °C for compositions (**a**)—F1, (**b**)—F3, and (**c**)—F5.

The scanning electron microscopy images of geopolymer binders (compositions F1, F3, and F5) prior to and after exposure to elevated temperatures are presented in Figure 18. It is evident from the analysis of the image of CBW-based geopolymer binder (F1) that a significant number of unreacted CBW particles are present within the geopolymer matrix prior to exposure to high temperatures (Figure 18a). After exposure to 200 °C, the microstructure is dominated by the formation of N-A-S-H gel, which appears to cover and integrate the CBW particles, indicating ongoing geopolymerization and gel development. Following exposure to 800 °C, the microstructure undergoes significant changes—due to sintering effects, where particles begin to fuse, contributing to a more consolidated structure.

The SEM image of the MKW–CBW blend-based geopolymer binder (F3) reveals a more homogeneous and compact matrix compared to composition F1 (Figure 18b). The presence of MKW appears to enhance the geopolymerization process, resulting in a denser gel phase with fewer visible unreacted particles. After exposure to 200 °C, the formation of N-A-S-H gel is evident, covering and binding the remaining CBW particles more effectively than in F1. Studies by Arunachelam et al. [91] have reported that N-A-S-H gel can be recognized in SEM micrographs as a dense, continuous matrix surrounding partially reacted or unreacted precursor particles. Upon heating to 800 °C, the microstructure shows signs of densification and sintering, with increasing porosity and smoother surfaces. In a similar study [92], specimens exposed to high temperatures (above 800 °C) exhibited higher porosity compared to those cured under ambient conditions. This increased porosity resulted in a reduction in mechanical strength.

The MKW-based geopolymer binder (F5) SEM image showed the presence of unreacted MKW particles embedded within the geopolymer matrix (Figure 18c). After exposure to elevated temperatures, some sintering and densification are observed, but residual unreacted particles remain visible, highlighting the influence of precursor composition on the final microstructure.

A subsequent comparison of the F1, F3, and F5 specimens after exposure (at a temperature of 800 °C) revealed that the F1 specimens exhibited the highest levels of residual strength. This phenomenon is attributed to the reinforcing effect of CBW particles, which contributed to a less porous and more homogeneous microstructure compared to the F3 and F5 specimens under elevated temperature conditions. Similar results were observed by Abd Ellatief et al. [93] and Ou et al. [94].

The relationship between the residual mechanical performance of the geopolymer binders and their microstructural evolution becomes evident when the strength data are interpreted alongside the XRD and SEM observations. The substantial compressive strength gains recorded at 200 °C (up to 61.8% for F1 and 52.3% for F3) in Figure 14a correspond directly to the increased formation and densification of the amorphous N-A-S-H gel. This is confirmed by SEM images showing enhanced gel coverage, as well as by FT-IR bands at 991–993 cm^−1^ that intensify due to secondary repolymerization. The stability of the crystalline phases, as detected by XRD up to 800 °C, also explains the moderate strength retention in CBW-rich mixes. Here, limited microcracking and the preservation of the quartz–feldspar phases maintain structural integrity. In contrast, the significant strength losses observed in MKW-rich compositions at 800 °C (up to 89.5% in F5) are consistent with XRD evidence of nepheline crystallization and SEM-identified increases in porosity and crack coalescence. This indicates a transition from a cohesive amorphous network to a brittle, thermally incompatible microstructure. Conversely, CBW-based binders either retain or improve in strength at 800 °C, thanks to thermally stable mineral phases and sintering-induced densification. SEM images show these to be fused, consolidated particle networks. Overall, these correlations demonstrate that the magnitude and direction of strength evolution are quantitatively governed by gel development, phase stability and microstructural densification or degradation under thermal exposure.

## 4. Conclusions

The study showed that the performance of CBW–MKW geopolymers is strongly governed by precursor reactivity, mixture chemistry, and thermal behavior.

- MKW increased heat evolution, reduced density, and delayed setting due to its higher reactivity. Increasing MKW content consistently improved compressive strength while lowering the Si/Al ratio. The MKW-only mixture (F5) reached 62.8 MPa at 28 days. This strength gain is linked to the formation of additional N-A-S-H gel, promoted by the lower Si/Al ratio. However, higher MKW required more alkalis, increasing porosity and reducing flexural strength.
- The CBW-only mixture (F1) showed the highest drying shrinkage (21.879 mm/m) due to low reactivity, excess water, and a high Si/Al ratio (4.89). Adding ≥25% MKW significantly reduced shrinkage by improving geopolymerization.
- F1 demonstrated the best water resistance (softening coefficient 0.92) because of its higher SiO_2_/Na_2_O ratio and lower Na_2_O and water contents. MKW-rich mixtures (F2–F5) showed higher water absorption and lower thermal stability due to increased porosity.
- All mixtures gained strength at 200 °C, especially MKW-rich binders (F4–F5), due to additional N-A-S-H gel formation. At 800 °C, MKW-rich binders lost strength because of high porosity and cracking.
- The microstructural results show that F1 stays crystalline and stable up to 800 °C, while F3 and F5 undergo phase changes that weaken their performance. XRD and FT-IR indicate that F1 preserves quartz, microcline, and albite, whereas F3 and F5 form nepheline and lose hydroxyl groups as temperature rises. SEM confirms these trends: F1 gradually sinters into a dense, cohesive matrix; F3 becomes more porous after heating; and F5 retains unreacted particles and significant porosity. After 800 °C, F1 remains the most consolidated and least cracked, matching its highest residual strength, while F3 and F5 show increased porosity and microcracking.
- Thermal shrinkage strongly influenced dimensional stability and residual strength. F1 exhibited the lowest shrinkage and mass loss at all temperatures, reaching only 21.081 mm/m and 7.5% at 800 °C, with no cracking. Its high Si/Al ratio (4.89) contributed to superior thermal stability. Increasing MKW content led to greater mass loss and thermal shrinkage. F5 showed the highest shrinkage (96.125 mm/m) and mass loss (18.5%) at 800 °C, accompanied by severe cracking and a major reduction in residual strength. These results confirm that the most rapid geopolymer shrinkage occurs between 650–800 °C and that CBW addition effectively reduces thermal shrinkage.
- Overall, CBW-based binders offer superior thermal stability up to 800 °C, while MKW enhances strength but reduces durability when used in high proportions. Further work should examine performance above 800 °C and evaluate the role of aggregates.

## Figures and Tables

**Table 1:** Oxide composition (wt.%) of ceramic brick waste (CBW) and metakaolin waste (MKW) [47].

Oxide	SiO_2_	Al_2_O_3_	Fe_2_O_3_	K_2_O	CaO	MgO	Na_2_O	TiO_2_	P_2_O_5_	SO_3_	Cl	Other	LOI	Total
CBW	70.49	13.24	4.72	3.72	3.67	2.13	0.88	0.69	0.15	0.10	0.03	0.18	0.85	100
MKW	55.28	30.90	0.90	0.69	2.85	0.87	7.46	0.53	0.10	0.14	-	0.28	1.93	100

**Table 2:** Formed compounds (wt.%) of ceramic brick waste (CBW) and metakaolin waste (MKW) [47].

Compound	Quantity, %							
	Quartz	Microcline	Hematite	Albite	Muscovite	Kaolinite	Anorthite	Amorphous
CBW	66.96	12.08	2.15	9.47	-	-	-	9.34
MKW	3.17	-	-	-	5.31	10.42	8.03	73.07

**Table 3:** Geopolymer binder compositions wt.%.

Mix No.	Precursor Materials		Alkaline Solutions		Essential Molal Ratios				Mix Design Parameters		
	CBW	MKW	NaOH	Na_2_SiO_3_	SiO_2_/Al_2_O_3_ (Si/Al)	H_2_O/Na_2_O	Na_2_O/Al_2_O_3_ (Na/Al)	SiO_2_/Na_2_O (Si/Na)	SSA^1^	AS/P^2^	W/S^3^
F1	100	0	14.0	21.0	9.79 (4.89)	15.45	0.68 (0.68)	14.42 (7.21)	497.96	0.35	0.21
F2	75	25	18.0	27.0	7.14 (3.57)	12.90	0.78 (0.78)	9.10 (4.55)	491.66	0.45	0.27
F3	50	50	22.0	33.0	5.55 (2.77)	11.67	0.85 (0.85)	6.54 (3.27)	485.36	0.55	0.32
F4	25	75	28.0	42.0	4.54 (2.27)	11.27	0.93 (0.93)	4.88 (2.44)	479.05	0.70	0.39
F5	0	100	32.0	48.0	3.77 (1.89)	10.76	0.95 (0.95)	3.95 (1.98)	472.75	0.80	0.43

SSA^1^—specific surface area, m^2^/kg; AS/P^2^—alkali solution to precursor (powder) ratio; W/S^3^—water to solids ratio.

**Table 4:** Fresh-state geopolymer density, setting time and workability parameters.

Mix No.	AS/P Ratio	Fresh Paste Density, kg/m^3^	Setting Time, min		Vicat Plunger Time to Bottom, s	Spread Diameter, mm
			Initial	Final		
F1	0.35	2031	150	205	0.61	180
F2	0.45	1945	220	275	0.63	180
F3	0.55	1875	290	345	0.55	185
F4	0.70	1765	330	390	0.43	190
F5	0.80	1694	540	600	0.41	190

## Data Availability

The original contributions presented in this study are included in the article. Further inquiries can be directed to the corresponding author.
